# Economic specialization and heterogeneous temperature-economy relationships suggest net costs of climate change in Europe

**DOI:** 10.1038/s41467-026-73341-4

**Published:** 2026-06-05

**Authors:** Manuel Linsenmeier, Ben Groom, Sefi Roth

**Affiliations:** 1 Department of Geography and Environment, London School of Economics and Political Science, London, UK; 2 Grantham Research Institute on Climate Change and the Environment, London School of Economics and Political Science, London, UK; 3 Climate School, Columbia University, New York, NY USA; 4 High Meadows Environmental Institute, Princeton University, Princeton, NJ USA; 5 Center for Critical Computational Studies, Goethe-University Frankfurt, Frankfurt am Main, Germany; 6 Dragon Capital Chair in Biodiversity Economics, LEEP Institute, Department of Economics, University of Exeter Business School, Exeter, UK

**Keywords:** Climate-change impacts, Environmental economics, Business and industry

## Abstract

Econometric studies of temperature and GDP imply that warming harms hot countries, benefits cool ones, and that a single globally optimal temperature exists. We show that such aggregate relationships mask substantial spatial and sectoral heterogeneity and can mislead mitigation and adaptation policy. Using administrative district-level data for Europe on Gross Value Added (GVA) and GDP growth, we estimate the contemporaneous effects of temperature at national, district, and industry scales. In contrast to earlier global studies, warmer-than-average years reduce growth in relatively cold districts (0–14°C) and raise it in warmer regions (>14°C), with the pattern reversing at the extremes (<0°C and >20°C). This U-shaped relationship implies an average effect across Europe of -0.19 percentage points on annual growth, rather than the +0.18 benefit reported previously. Under RCP4.5, annual growth falls by 0.20 to 1.24 percentage points by 2070–2099, highlighting local temperature optima and heterogeneous vulnerabilities both within countries and across regions and sectors.

## Abstract (web summary)

Europe’s temperature-growth link is U-shaped; not globally uniform: warming lowers growth in many colder districts, raises it in some warmer ones, and reverses at extremes. Overall, Europe faces net growth losses under climate change, with strong sectoral and regional heterogeneity.

## Introduction

Economic analysis on the effect of annual mean temperature fluctuations on the economy suggests that high temperatures are negatively linked with GDP growth^1–3^. These empirical studies point to a non-linear relationship between temperature and economic performance, whereby aggregate impacts on growth are negative in regions with already higher average temperatures, yet are beneficial to regions with lower temperatures. These aggregate effects are argued to be relatively similar across countries, and supported by certain micro-economic studies of individual economic behaviours and activities, such as crime or arable agriculture^1,4^. However, the global studies that have given rise to this inverted U-shaped temperature-economy relationship estimate temperature-economy relationships that reflect the average effect of heterogeneous regional temperature-economy relationships. Furthermore, country-level, economy-wide temperature-economy relationships reflect the net effect of temperature on productivity across the constituent sectors of the economy and across different sub-national regions^5^. In each case, important sectoral and geographic heterogeneity, for which there is evidence at the micro-level for outcomes such as wages^6^, health^7,8^, and recreation^9^, is overlooked. The heterogeneity in the response of GDP to temperature shocks is, however, critical to inform efficient climate adaptation and mitigation policies.

Just as economic stabilisation policies are tailored to the vulnerabilities of the different sectors of the economy^10^, policies geared towards adapting to, and decoupling economic activity from climate change should also be informed by the vulnerabilities of specific sectors in different regions^11,12^. Disentangling the pattern of economic costs, both spatially and across the economy, can also inform efforts to mitigate climate change by providing more accurate measures of economic damages. A more detailed understanding of the link between temperature and economic activity across different sectors and sub-national geographies: an economic geography of climate change, is therefore required^13^.

Here we present the results of a comprehensive analysis of the pattern of the temperature-economy relationship for Europe using exhaustive national accounting data at the aggregate and sectoral level for sub-national districts, combined with weather data of the same granularity. We use two main sources of data in our analysis. Economic data is taken from EUROSTAT at the level of NUTS-3 administrative districts in Europe. Importantly, this data is available in aggregate and by industry. Economic production is measured as Gross Value Added (GVA), which is defined as Gross Domestic Product (GDP at basic prices) minus intermediate consumption (inputs at producer prices). For robustness, we replicate our results with data on aggregate (not sectoral) GDP at both the national and subnational levels. For temperature and precipitation, we use high-resolution reanalysis data from the European Centre for Medium-Range Weather Forecasts (ECMWF). Overall, our final dataset combines year-to-year fluctuations of temperature and precipitation with district-level annual GDP and GVA in six exhaustive industry groups and covers over 1500 individual districts in 33 European countries between the years 1980 and 2019.

To identify the link between temperature and economic output, we use regression methods for panel data. Specifically, we estimate Fixed-Effects (FE) models that control for unobservable characteristics at the district-level and for each year. We follow previous studies^1–3^ and use the growth rate of GVA (or GDP) as our dependent variable, which allows us to identify changes in productivity and to address possible concerns about non-stationarity of the time series^3,14^. The temperature-economic growth relationship is modelled with either polynomials or more flexible splines of annual mean temperature. In the main specification, our model also includes linear time trends by district. In additional robustness checks, we also estimate models with bins of annual mean temperature and with different time controls. To examine the spatial heterogeneity of the relationship, we estimate models with country- and district-specific coefficients of temperature. We generally focus on the contemporaneous effect of temperature shocks on growth rates in the same year. Our principal contributions are twofold. First, we provide a comprehensive, Europe-wide estimate of how annual mean temperature (not extreme events^15^) affects aggregate income across space and economic sectors. By disaggregating the effects at the NUTS-3 district and sectoral levels, we reveal substantial variation in temperature sensitivity across regions and industries. Second, we find that the aggregate (global) relationship between temperature shocks and GDP, which exhibits an inverted U shape, is not robust in the European case. The findings indicate the unique structure of aggregate, spatial, and sectoral costs that can emerge from more granular analysis, and the need for bespoke climate adaptation strategies in Europe.

## Results

**Fig. 1:**
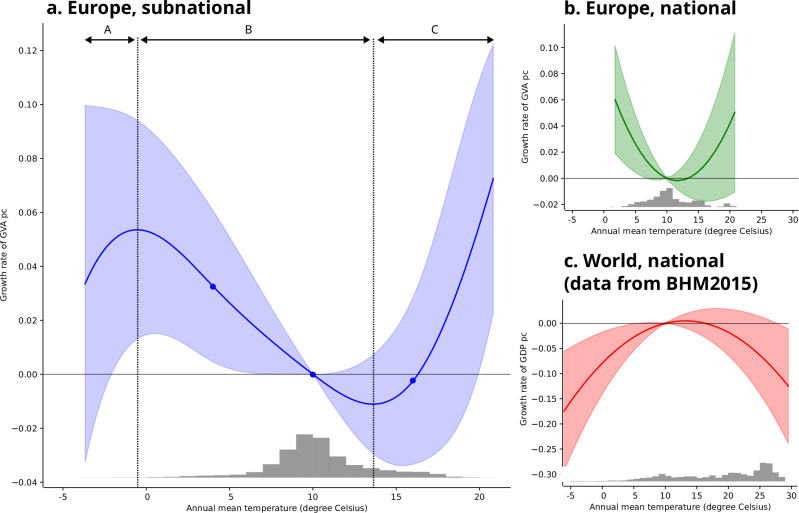
Main estimates and the number of observations in the data. Lines show the central estimates, uncertainty ranges in light colour show the 95% confidence intervals based on the estimated standard errors. **a** Effect of annual mean temperature on the growth rate of GVA per capita in districts in Europe. The response curve suggests a positive effect of higher temperatures over the intervals A and C and a negative effect over the interval B. **b** Effect of annual mean temperature on the growth rate of GVA per capita in countries in Europe. **c** Effect of annual mean temperature on the growth rate of GDP per capita in countries in a global sample using replication data of Burke et al. (2015)^1^.

Our main estimate shows an approximately quadratic relationship between annual mean temperature and economic growth, with negative effects of warmer-than-average years in cold regions (0 to 14 °C) and positive effects in warm regions (above 14 °C) (Fig. 1a, b; marginal effects in Supplementary Fig. 8). Importantly, these results are in contrast to previous work using global samples of countries^1^ and sub-national regions^3^, which suggested an optimal temperature of 13^1^ and 5–10 °C^3^, respectively (Fig. 1c). Our results suggest significant costs to higher temperatures in moderately cold regions. Moreover, our U-shaped temperature-economy relationship questions the idea of a globally optimal temperature. Rather, the findings point to the existence of local optima, driven by a combination of location, composition/specialisation of the economy, and possibly institutions. We investigate this heterogeneity in detail below.

Our results are only in line with earlier studies at the extremes of the European climate: for very cold and very warm regions. Specifically, we find that for very cold regions (average temperature less than 0 degrees Celsius), warmer-than-average years increase economic growth. Conversely, in very warm regions (warmer than 20 degrees Celsius), our most flexible models suggest that warmer-than-average years may have a negative effect (Supplementary Fig. 9b, e, f). Admittedly, the geographical support for these temperature extremes is naturally much narrower in Europe than in a global sample of countries (particularly at the right tail of the temperature distribution). The negative effect on cold districts accords qualitatively with non-significant results in prior studies^1^.

To understand why our main results are different from previous studies, we examine whether these discrepancies can be explained by differences in the economic and weather data. We therefore estimate the same relationship at the national (rather than district) level using a polynomial specification and find that the results are similar (Fig. 1b). Similar results are also obtained when we use replication data from^1^ (Supplementary Fig. 10b) and when we use our data with GDP instead of GVA (Supplementary Fig. 11b). These findings suggest that the differences are primarily due to our geographical focus on Europe and to some extent our more spatially granular data. Overall, districts and countries in Europe appear to respond differently to a warmer-than-average year than the results from a global sample suggest. Documenting this geographical and jurisdictional heterogeneity is important for climate-related policy.

To further establish the robustness of the overall relationship between temperature and economic growth in Europe, a number of robustness tests are undertaken. We first estimate models with different specifications of polynomials and splines of annual mean temperature (Supplementary Fig. 9b, c) and with bins of annual mean temperature (Supplementary Fig. 9e, f). Furthermore, we estimate models with different time controls (no trends, quadratic district-specific trends)(Supplementary Fig. 11c, d). We also estimate our model using a balanced panel of districts that includes only the last 12 years of data (Supplementary Fig. 11e). In an additional exercise, we include one by one 36 alternative measures of rainfall in our model^16^(Supplementary Fig. 12a), also allowing for heterogeneous effects (Supplementary Fig. 12b). We find that our main results are less precise without controlling for time trends and generally more negative for the balanced panel, but overall the pattern of the estimated effects remains the same. In another robustness check, we use only the “surprise component” of the temperature time series^17^ and again recover essentially the same non-linear effects across Europe (Supplementary Fig. 13).

The estimated effect of warmer-than-average years on economic growth is economically significant, too. To quantify its magnitude, we compute the marginal effect of an increase in temperature by 1 degree Celsius over the observed range of temperature levels in Europe (Supplementary Fig. 8). Between 0 and 14 degrees Celsius, where the marginal effect is negative, we find that such a temperature shock lowers economic growth by, on average, about −0.45 percentage points. Above 14 degrees Celsius, where the marginal effect is positive, the marginal effect is on average +1.2 percentage points and peaks around 20 degrees Celsius at +1.9 percentage points. Over most of the range, the estimated effects are statistically significant (Supplementary Fig. 8). For example, at 5 degrees Celsius, the estimated effect is -0.59 percentage points (95% CI is [−1.04, −0.14]), and at 20 degrees Celsius it is +1.9 percentage points ([1.3, 2.5]). Additional analysis suggests that the drop in GVA is persistent in cold regions, but the cumulative effect is similar to the contemporaneous effect, somewhat sensitive to the model specification in warm regions (Supplementary Table 2 and Supplementary Fig. 14).

**Fig. 2:**
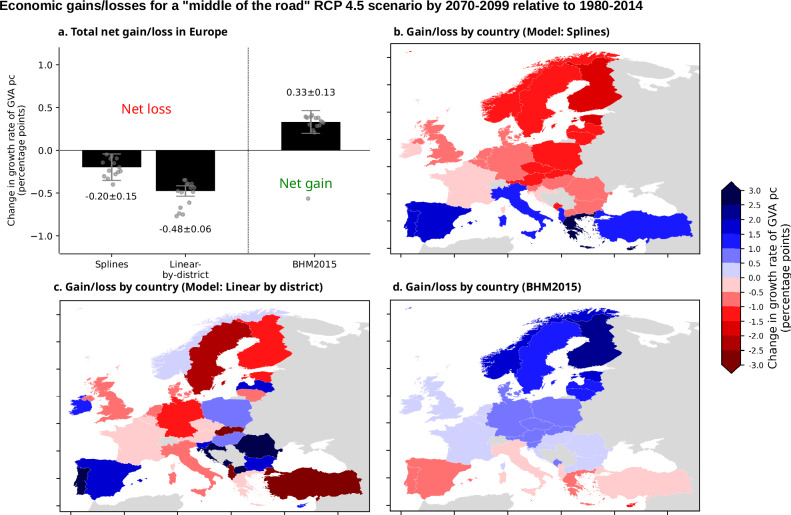
Effect of future climate change on average annual economic growth for an RCP4.5 scenario by the end of the century (2070–2099 versus 1985–2014). Projections for alternative specifications of the econometric model (Methods). All figures show the median of the total costs obtained from *n* = 15 individual climate models of the CMIP6 ensemble. Base maps adapted from World Bank Official Boundaries under a Creative Commons licence CC BY 4.0. **a** Overview over total costs (net gain/loss) for three model specifications and data sources. Black bars show median of climate model ensemble, grey bars the 95 % confidence interval of the median, and grey dots the results for the individual climate models; result for the climate model CMCC-CM2-SR5 are not shown because outside the range; see Supplementary Table 3 for the full distribution and additional model specifications. **b** Costs by country based on our model with splines shown in Fig. 1a. **c** Costs by country based on our model with district-specific coefficients (for visualisation, the costs aggregated to countries). **d** Costs by country based on the estimates of Burke et al. (2015)^1^.

For ease of interpretation, we quantify the effect of a 1 degree Celsius warmer than average year on total economic growth for the whole of Europe. These estimates are obtained by calculating for every district the predicted growth rates for a year with the historical average annual mean temperature and for a year with the same temperature +1 degree Celsius, taking the difference between the two, multiplying it by the GVA of that district, and then summing these products over all districts in Europe. Net of costs and benefits, our results suggest that a uniformly 1 degree C warmer-than-average year reduces economic growth in Europe by −0.19 ([−0.33, −0.07]) percentage points. Our main results take a more realistic approach to the estimation of the costs of future climate change by undertaking the same calculations but based on actual CMIP6 climate model projections for the RCP4.5 scenario for the end of the century (2070–2099) relative to the historical period (1980–2014). We find net costs of climate change in Europe in terms of a reduced average annual growth rate of about −0.20 ([−0.35, −0.05]) percentage points for Europe as a whole and −0.48 ([−0.54, −0.42]) for models that allow for heterogeneity by district. See Fig. 2a. Importantly, both of these results are in contrast to previous work. To illustrate this difference, we use the estimates of ref. ^1^ obtained from a global sample of countries and applied them to our sample of districts in Europe. The result of this exercise is also presented in Fig. 2a and suggests a net positive effect of about +0.33 ([0.20, 0.46]) percentage points.

For visualisation, we also aggregate our cost estimates of individual districts to the level of countries. Using our splines and linear models, we show that most countries in the center and North of Europe are projected to have negative effects, while most countries in the South are projected to have positive effects of above-average temperatures (Fig. 2b, c). Again, this is in contrast to previous work that suggests that countries in the centre and North benefit from warmer temperatures (Fig. 2d). We again corroborate our results with extensive robustness tests (Supplementary Fig. 16 and Supplementary Table 3). While the direction of the effect remains consistent, the projected costs of future climate change are substantially larger when using a balanced panel of the most recent 12 years of data (−0.72 [−0.96, −0.48]) or when estimates are conducted at the country level (−1.24 [−1.32, −1.16]). For robustness, we estimate a model inspired by ref. ^17^ with lagged temperatures, which yields very similar results (Supplementary Fig. 11).

**Fig. 3:**
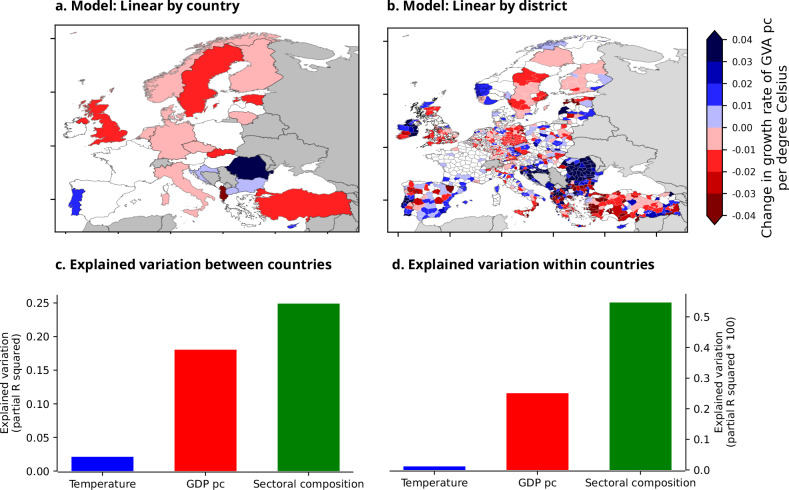
Marginal effect of an increase in annual mean temperature on economic growth obtained with alternative model specifications based on our sample of districts in Europe. Base maps adapted from World Bank Official Boundaries under a Creative Commons licence CC BY 4.0. **a** Country-specific coefficients of annual mean temperature. **b** District-specific coefficients of annual mean temperature. **c** Explained variation of marginal effect between countries. **d** Explained variation of marginal effect within countries. In (**a**, **b**), the white regions indicate regions/districts where the coefficients were not statistically significant at the 5% level.

The temperature-GVA relationship in Fig. 1 reflects the net effect of temperature on aggregate productivity but is silent on composition. We unpack these aggregate level impacts on GVA spatially and by the component sectors of the economy. We first examine heterogeneity in space. To do so, we estimate models in which the effect of a warmer-than-average year can differ by country and by subnational district regardless of their average climate (see Methods section for more details). Motivated by our earlier results that suggest similar total costs for Europe (Fig. 2a), but also substantial variation in costs among countries with similar annual mean temperature (Fig. 2b, c), we further unpack the heterogeneity in the marginal effect of temperature on economic growth in Fig. 3, where countries and districts with insignificant estimates are shown in white.

In the analysis by country, we find negative marginal effects of higher-than-average temperatures in most of Northern Europe up to a latitude of about 50 degrees North (Fig. 3a). Further South, we find mixed results. Some countries, like Italy and Turkey, are negatively affected by higher-than-average temperatures, while others, like Portugal and Cyprus, benefit from higher-than-average temperatures. Indeed, some countries, like Spain and France, appear to be unaffected (resilient) at the aggregate level.

The analysis disaggregated by district reveals a great deal of heterogeneity. Figure 3b shows our district-specific coefficients of annual mean temperature. Two important conclusions can be drawn from this analysis. First, there is heterogeneity across districts within countries in their response to an increase in annual mean temperature. Second, the country-level aggregate effects stem from averaging positive and negative effects across districts within a country. This finding has potential implications for adaptation policies, since country-level estimates of impacts could provide a misleading picture of local impacts. For instance, concluding from a national level analysis that temperature deviations have no effect on the economy, as seen in Spain and Ireland in Fig. 3, ignores important sub-national heterogeneity. Similarly, large aggregate positive (e.g. Portugal) or negative (e.g. UK or Sweden) effects of temperature deviations can be largely driven by specific sub-national regions.

Fluctuations in annual mean temperature from year to year represent the net effect of the temperature of different seasons. We find that in about half of the countries in our sample, fluctuations in annual mean temperature are to a similar extent the effect of temperature in summer and temperature in winter (Supplementary Figs. 5–7). In most of the remaining countries, fluctuations in annual mean temperature are primarily due to warmer or colder than average winters. Only in Albania and Macedonia the annual mean temperature appear to be primarily determined by temperature in summer. This geographic variation in annual mean temperature fluctuations can potentially explain some of the heterogeneity in economic responses, as we illustrate in the discussion of economic mechanisms further below. Another important question arising from this variation is to what extent the changes in the distribution of daily temperatures associated with past fluctuations in annual mean temperature relate to changes in the distribution of daily temperatures that are associated with future warming, and how this may affect the expected costs of future warming. In an additional robustness test, we explore this question by using degree-day models rather than models of annual mean temperature. We find that this approach yields no statistically significant estimates of the costs of future warming (Supplementary Fig. 16 and Supplementary Table 3).

The estimation of degree-day models also allows us to address the more theoretical question about whether our results are indicative of location-specific optimal temperature levels, rather than a globally optimal temperature. Previous work estimated a non-linear model relating annual mean temperature to economic growth rates based on a pooled sample of countries. This resulted in evidence consistent with a globally optimal annual mean temperature of around 13 degrees Celsius^1^. We explore this question by estimating one degree-day model for every country and then extracting the daily temperature level with the highest predicted growth rate. We find a large variation in these “optimal” temperature levels across countries, ranging from −10 to 25 degrees Celsius (Supplementary Fig. 17). When we correlate the optimal daily temperature with the average annual mean temperature of a country, we do not find any significant correlation. In sum, we interpret this as suggestive evidence for relatively idiosyncratic response curves by country, justifying the use of models that allow for heterogeneity by country or even by district.

We next examine more comprehensively which factors determine how the economy of a district or a country in Europe responds overall to a warmer-than-average year. We consider several moderating factors simultaneously, namely the average climate, income per capita, and sectoral composition. Our results suggest that most variation between countries can be explained by the sectoral composition, followed by income per capita and annual mean temperature levels (Fig. 3c). The importance of sectoral composition relative to the other factors is even more pronounced within countries (Fig. 3d).

**Fig. 4:**
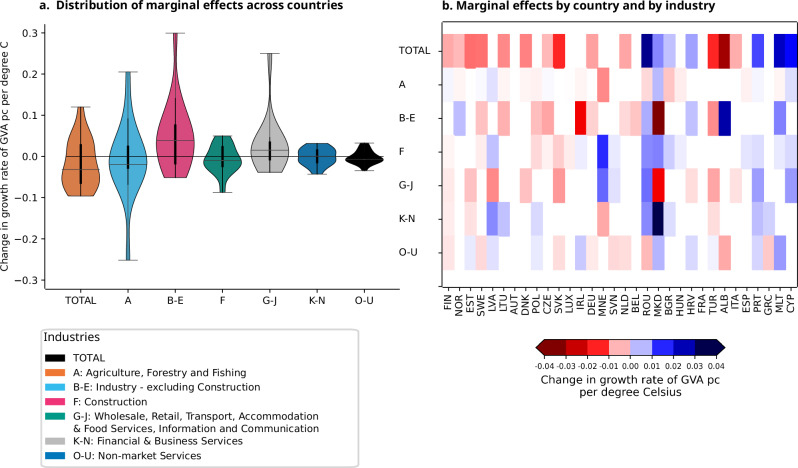
Marginal effect of an increase in annual mean temperature on the growth rate of GVA in six different industry groups. The sample here excludes the United Kingdom, which uses a different sector disaggregation. **a** Distribution of country-specific coefficients of annual mean temperature for total GVA and GVA in six industry groups over all 32 countries in the sample. Horizontal lines indicate mean values, boxes show inter-quartile ranges, and whiskers the min/max of the distributions. **b** Country-specific coefficients of annual mean temperature for total GVA and GVA in six industry groups for individual countries.

The within-country results motivate further analysis of heterogeneity by examining the temperature-GVA relationship by industry group. In particular, we study how yearly temperature fluctuation affects GVA growth in six broad groups that together make up total GVA: Agriculture, Industry, Construction, Financial and Business Services, Non-market Services, and a broad group including Wholesale, Retail, Transport, and other services (in the following referred to as Trade). Recall, GDP data is not available by sector. For every industry group, we find districts with positive effects of warmer-than-average temperature and districts with negative effects (Fig. 4a). The largest number of districts with negative effects are found for Industry, Agriculture, and Trade. Positive effects are most frequently found for Construction. This relatively large sensitivity of Agriculture, Industry and Construction to weather is consistent with the relatively large influence of seasonality on these sectors^18^. Some of these results concerning GDP/GVA growth accord with previous cross-sectoral studies (e.g. agriculture), while others differ (e.g. financial and other services are positive here)^19^. That temperature shocks hurt some industries, and not others, is also found in the analysis of financial returns and productivity at the firm level^20^, albeit sometimes close to zero even for sectors that are in principle high-risk^21^.

Overall, we find considerable geographical variation of marginal effects within every industry group. We therefore also differentiate by country (Fig. 4b) and find large variation. In many cases, countries with similar climates show different responses of a specific sector to a warmer-than-average year. However, there are some overall patterns, albeit each with its exceptions. In Industry and Construction, negative effects of warmer temperatures are mostly observed in colder countries. Trade (G–J) and Agriculture are other sectors that exhibit mostly negative responses in cold countries. There seems to be even more variation in warmer countries, with Construction as the only sector with a consistently positive response to higher temperatures. We add further texture to the heterogeneous effects across sectors and countries by splitting the estimation shown in Fig. 4b into responses to temperature shocks in summer (JJA) and winter (DJF) (Supplementary Fig. 19). This analysis shows that the impact of temperature shocks is rather consistent throughout the year in most sectors, with the largest variation between the seasons in the response to temperature in Trade (G–J). The results also suggest that, overall, it is especially warmer-than-average winters that are harmful for economic activity, with less consistent results for warmer-than-average summers. This pattern is particularly pronounced in the coldest countries.

While the nature of our data prevents us from causally identifying the underlying mechanisms for this heterogeneity, we posit some possible explanations broadly classified into two categories of response to warming: physical and behavioural. On the physical side, in cold regions of Europe, warmer temperatures in winter have been found to be detrimental to growth (e.g. crops) in agriculture^22^. Furthermore, warmer-than-average temperatures have generally been found to negatively affect fisheries^23,24^. In construction and mining, we hypothesise that warmer-than-average temperatures around the freezing point detrimentally affect economic activity through increased costs of, e.g. pumping meltwater or subsidence^25–27^. Furthermore, warmer-than-average temperatures can also cause damage to transport infrastructure in Northern regions^28^. On the behavioural side, warmer-than-average temperatures in cold regions are likely to reduce the energy demand of the economy, thereby reducing revenues in Utilities. Finally, in industries with most work taking place indoors, such as Trade and Other Services, warmer-than-average temperatures in cold regions could affect working hours similarly to rainfall and very hot days by changing the opportunity costs of work versus leisure^29,30^. For these and related reasons, a number of studies have shown that temperature shocks also affect activity through labour productivity (see, e.g.,^31,32^). Further research to explain how these potential mechanisms translate into the precise pattern of spatial and sectoral heterogeneity would be a fruitful next step in the analysis.

## Discussion

The prior empirical evidence on the global relationship between economic activity and temperature variation suggests that higher-than-average temperatures in warmer-than-average locations are costly, but the same shocks in cold areas can be economically advantageous^1^. Such findings underpin a common narrative on the expected costs of climate change and their spatial distribution, and inform national and regional adaptation strategies. The EU strategy on the adaptation to climate change^33^ largely reflects this narrative, using examples from climate change ‘hotspot’ areas in Southern Europe and the Mediterranean as exemplars of the geographical incidence of climate damages and the need for strategic interventions on climate change adaptation. The results of this paper invert this narrative in the European context and show that positive temperature shocks can be costly to many cold regions of Europe.

These findings are of general interest and show the importance of detailed spatial and sectoral analysis, and a regional focus for estimating heterogeneous temperature-economy relationships. The findings can inform the broader efforts to assess the Social Cost of Carbon and the desirability of meeting the 1.5 °C Paris target using Integrated Assessment Models, via updated estimates of damage functions^34^. In this regard, our results on regional and sectoral heterogeneity also make the unequal geographical distribution of the costs of climate change explicit. More specifically, the results can inform the EU strategy to forge a climate-resilient Europe by 2050^33^. We provide an up-to-date picture of the potential socio-economic impacts of climate change, fulfilling the EU strategy’s desire to ‘anchor smarter adaptation in the latest science’. Our illustration of the spatial pattern of the temperature-economy nexus could also inform EU-wide adaptation policies, such as the coordination of genetic diversity sharing in agriculture, or the coordination of regional programmes of investment in, e.g., adaptation innovation. The finding that the costs of temperature shocks are also felt in colder regions can also help to prioritise scarce regional development, infrastructure and adaptation funds to more vulnerable locations and sectors where the benefits will be higher. More broadly, our spatially explicit results help to identify synergies and trade-offs between adaptation to climate change and the objectives of regional development in Europe.

Ultimately, the higher costs of temperature shocks in cold regions, where warming associated with climate change is expected to be greatest, reflect a socio-economic specialisation, both behavioural and structural, around their colder climates. In this sense, the temperature-economy relationship estimated here suggests that the concept of a globally optimal temperature is misplaced. Contrary to previous findings, from the perspective of income, local temperature optima seem more likely. In colder regions of Europe, some like it cold.

The paper illustrates that care is needed in evaluating the impacts of temperature change and drawing broad conclusions about how the costs and benefits of climate change are distributed across different geographies. Considering Europe separately from the rest of the world has provided important new insights. Future research can further illuminate the costs of climate change in cold regions of the world outside Europe. This will add more evidence on the mechanisms through which warmer-than-average years can reduce economic production in certain industries. Furthermore, using disaggregated data allowed us to study geographic and industry heterogeneity in great detail, but the relatively few years of observations prevent us from studying the dynamics and persistence of temperature shocks. Our robustness tests suggest that the non-linear patterns are similar for cumulative effects. Future research may further explore the temporal dynamics and persistence, building on the existing literature^17,31,32,35^.

Similarly, our focus on the response of local economies to local temperature shocks allows us to study heterogeneity in great detail, but we do not attempt to quantify any indirect costs of future climate change to a location due to its trading relationship with the rest of the world, which may be substantial^36,37^. Other research questions arising from our results concern the possibilities of adapting to gradual changes of temperature in the long-run, which might be underestimated or overestimated in our results. The role of specialisation, and the spatial and sectoral linkages through which temperature effects operate, are also obvious avenues for future research. While we have demonstrated the heterogeneous effects across space and sectors, we are limited in what we can say about the precise mechanisms that lead to the spatial and sectoral patterns we observe. As more granular data becomes available, new insights into the challenges of climate change can be gained from the geographically explicit analysis found in this paper, and a broader evidence base developed to inform policy. Detailed analysis of individual countries, especially disaggregated by industry and season (e.g.^19,38^), can complement regional and global analysis. If the objective is to better understand the impact of climate change, and hence better prepare and adapt, mechanisms relating to labour productivity, investment, the response of infrastructure and health appear to be primary areas of interest for future research. Future research should also bear in mind that GDP and GVA do not capture all of the impacts of climate change on long-term human well-being and the biosphere.

## Methods

### Data

We use data on temperature and precipitation from high-resolution reanalysis provided by the ECMWF (ERA5-Land). The data has a resolution of about 9 km. We aggregate it to administrative districts using gridded population data from the Gridded Population of the World dataset in version 4^39^ (Supplementary Fig. 1).

We complement the climate data with data on GVA by industry from EUROSTAT provided as part of the ARDECO database. The data is available at the level of NUTS-3 administrative districts in Europe (NUTS-2 for Turkey). We drop all overseas territories, restricting the dataset to geographical Europe (Supplementary Fig. 2). We complement these data with data on population from the same (NUTS-3) sources. The data uses the NACE v2 industry classification with a breakdown of total GVA into 6 industry groups. We complement this dataset with regional GVA from the UK Office for National Statistics, since data for the UK is no longer provided by EUROSTAT.

The economic data have different temporal coverage for different countries. Exploratory analysis also reveals that for some countries, the first years in the dataset are temporally interpolated since the growth rates of several districts of those countries are (almost) identical (Supplementary Fig. 3). We drop those years from our data as indicated in Supplementary Fig. 3. To improve the balance of our final panel dataset, we then drop spatial units with less than 10 observations in time. In the final sample, temporal coverage ranges from 12 years in Albania to 40 years in e.g. France (Supplementary Fig. 4). In a robustness test, we use a balanced panel that includes only the last 12 years of each location. Descriptive statistics are provided in Supplementary Table 1.

Future climate projections are based on the CMIP6 model ensemble. We use the bias-corrected Global Daily Downscaled Projections from NASA^40^. We download future (2070–2099) and historical (1985–2014) simulations for the RCP4.5 scenario for each climate model. Some members of the CMIP6 ensemble are considered as “too hot”^41^. We follow the recommendations and data provided by ref. ^41^ and select all models whose equilibrium climate sensitivity lies within the “likely” range according to the IPCC. This yields a final ensemble of 15 models (Supplementary Table 3).

### Econometric framework

We estimate fixed-effects models with the growth rate of GVA per capita (either total or for a specific industry) as the dependent variable. In robustness checks, we also use the growth rate of GDP per capita as the dependent variable. As independent variables, we use polynomials, splines, and bins of annual mean temperature. We also include precipitation and time trends. The temporal frequency of our variables is years. This means that we exploit exogenous fluctuations of temperature from year-to-year for the identification of plausibly causal effects of temperature on economic activity.

Our main model can be written as

1 $${g}_{i,y}=f\left({T}_{i,y,d};{{{\boldsymbol{\beta }}}}\right)+h\left({P}_{i,y,d};{{{\boldsymbol{\gamma }}}}\right)+{\alpha }_{i}+{\theta }_{y}+y{\xi }_{i}+{\varepsilon }_{i,y}$$

where observations are indexed by administrative districts *i* and years *y* and days *d*, growth rate of GVA per capita *g*, temperature *T*, precipitation *P*, vectors of parameters *β* and *γ*, and district and year fixed-effects *α*_*i*_ and *θ*_*y,*_ respectively. In our main specification, we also include district-specific time trends *ξ*_*i*_. We conduct robustness tests for which we drop the time trends or include quadratic district-specific time trends (Supplementary Fig. 11).

In most model specifications, the function *f*(. ) is a function of annual mean temperature ${\overline{T}}_{i,y}=\frac{1}{{N}_{d}}{\sum }_{d=1}^{{N}_{d}}{T}_{i,y,d}$. In our main specification, *f*(. ) is represented by cubic splines with three knots. We also estimate a model with five knots, with a quadratic polynomial, and with bins of 2 degrees and 4 degrees width. In our main specification, *h* is a quadratic polynomial function of the annual total precipitation ${\overline{P}}_{i,y}={\sum }_{d=1}^{{N}_{d}}{P}_{i,y,d}$. In robustness tests, we use all 36 indicators of annual rainfall tested by ref. ^16^ (including the number of days on which rainfall exceeds certain thresholds, as well as the total rainfall on those days) (Supplementary Fig. 12). We also undertake the analysis with a cubic-spline for precipitation. The results remain robust, and we do not report them here.

To examine heterogeneity of the estimated effects, we estimate models in which we allow for the effect of annual mean temperature to differ by district or by country. This means that the function $f={\sum }_{i}{\overline{T}}_{i,y}{\beta }_{i}$ to obtain district-specific linear coefficients *β*_*i*_ and $f={\sum }_{c}{\overline{T}}_{i,y}{\beta }_{c(i)}$ to obtain country-specific linear coefficients, whereby the function *c* maps districts *i* to countries. At the level of countries, we also estimate a quadratic-by-country model with $f={\sum }_{c}{\overline{T}}_{i,y}{\beta }_{1,c(i)}+{\left({\overline{T}}_{i,y}\right)}^{2}{\beta }_{2,c(i)}$. We proceed analogously to estimate linear models with country-specific and linear models with district-specific coefficients of rainfall (Supplementary Fig. 12).

For an additional robustness test with daily mean temperature, we estimate a similar model with the number of days within certain intervals of daily mean temperature, also referred to as a degree-day model. We again allow for heterogeneity at the country level. This means that the function $f={\sum }_{k}{b}_{i,y}^{k}{\beta }_{c(i)}^{k}$ whereby the variables ${b}_{i,y}^{k}$ count the number of days on which daily mean temperature *T*_*i*,*y*,*d*_ falls within bin *k* in district *i* in year *y*.

We also check robustness to certain time-series properties of both temperature and GDP that can bias estimates of the effect of weather on economic growth. To show robustness against a dynamic bias, we include a lagged dependent variable in our model, *g*_*i*,*y*−1_ with coefficient *ρ* (Supplementary Fig. 11f, h). We also include three lags of annual mean temperature ${\overline{T}}_{i,y-l}$ (Supplementary Fig. 11g, h). Furthermore, we implement the solution proposed by ref. ^17^. We first regress annual mean temperature on its own lags, allowing the coefficients of the lags to vary by country to allow for regional heterogeneity in the dynamics of temperature: ${\overline{T}}_{i,y}={\sum }_{l=1}^{3}{\beta }_{l,c(i)}{\overline{T}}_{i,y-l}+{\alpha }_{i}+{\theta }_{y}+{\widetilde{T}}_{i,y}$. We are here interested in the residual ${\widetilde{T}}_{i,y}$, which we refer to as the “surprise” component of the temperature time series. To account for the moderating effect of the mean climate on the effect of temperature shocks on economic growth, we estimate a linear-by-interval model, interacting the shocks with dummies for intervals of climatological annual mean temperature *δ*_*k*_: $f={\sum }_{k}{\delta }_{k}{\widetilde{T}}_{i,y}{\beta }_{k}$. For ease of comparison, we estimate the same linear-by-interval model also with the original annual mean temperature data as shock, i.e. $f={\sum }_{k}{\delta }_{k}{\overline{T}}_{i,y}{\beta }_{k}$ (Supplementary Fig. 13).

Our analysis focuses on the contemporaneous marginal effect of a temperature shock on economic growth in the same year, defined as $\frac{\partial {g}_{i,y}}{\partial {T}_{i,y}}$. In additional analysis, we study the cumulative effect of a temperature shock in year *y* = 0 on log GDP in year *y* = *Y*, defined as $\frac{\partial \log {{{{\rm{GDP}}}}}_{i,Y}}{\partial {T}_{i,0}}={\sum }_{y=0}^{Y}\frac{\partial {g}_{i,y}}{\partial {T}_{i,0}}$. We calculate the cumulative effect by estimating a distributed-lags model with lags of temperature. For parsimony, we focus on the polynomial model (Supplementary Fig. 9c) as the simplest model that can produce our main finding on the spatial heterogeneity of temperature effects and because it is the model used in ref. ^1^. We estimate models with up to *L* = 5 lags: $f={\sum }_{l=0}^{L}\left({\overline{T}}_{i,y-l}{\beta }_{1,l}+{\left({\overline{T}}_{i,y-l}\right)}^{2}{\beta }_{2,l}\right)$. We then calculate the cumulative marginal effect as ${\sum }_{l=0}^{L}\left({\beta }_{1,l}+2\overline{T}{\beta }_{2,l}\right)$ (Supplementary Fig. 14 and Supplementary Table 2). In an additional robustness test we allow for more flexibility by estimating a linear-by-interval model with dummies for intervals of climatological annual mean temperature ($f={\sum }_{l=0}^{L}{\sum }_{k}{\delta }_{k}{\overline{T}}_{i,y-l}{\beta }_{k,l}$; see also preceding paragraph) with lags and calculate the cumulative marginal effect of each interval *k* as the sum of the corresponding coefficients across lags as ${\sum }_{l=0}^{L}{\beta }_{k,l}$ (Supplementary Fig. 14). For the dynamic model specifications we calculate dynamic cumulative effects that account for the propagation of growth shocks through the lagged dependent variable. To do so, we multiply the cumulative effect by the limit of the geometric series, $\frac{1}{1-\rho }$, with the lag coefficient *ρ* (see also ref. ^2^) (Supplementary Fig. 15).

For projections of the effect of climate change on GDP for the RCP4.5 scenario (2070–2099 versus 1985–2014), the costs are quantified as changes in the average annual growth rate between the two periods and shown in percentage points (see also Supplementary Table 3). We use our estimated models to calculate the estimated annual growth effect ${\widehat{g}}_{i,y}$ for every year of the historical and future period. We then subtract the average annual growth effect of the historical period from the average annual growth effect of the future period. We do this for each climate model separately and then calculate the median of the final cost estimate.

For consistent results at the level of countries and districts, districts are weighted with their share of national GVA. To account for heteroskedasticity, serial autocorrelation, and spatial autocorrelation of the error term, we cluster standard errors at the level of countries. We also try clustering at the second administrative level and using Conley HAC standard errors, but since these tend to yield smaller standard errors, we decide to choose the most conservative clustering method.

### Reporting summary

Further information on research design is available in the Nature Portfolio Reporting Summary linked to this article.

## Supplementary information

Supplementary Information

Peer Review file

Reporting Summary

## Data Availability

The original data are publicly available at no cost. See Methods Section for individual sources. The data for replication are available here: https://zenodo.org/records/19691941^42^.

## Code availability

The code for replication is available here: https://github.com/mlinzze/climate-europe^42^.
