# Supplementary material for: Economic specialization and heterogeneous temperature-economy relationships suggest net costs of climate change in Europe
Source: Nat Commun. 2026 Jun 5;17:7194. doi: 10.1038/s41467-026-73341-4 (PMC13396729; doi:10.1038/s41467-026-73341-4)

Economic specialization and heterogeneous temperature-economy relationships suggest net costs of climate change in Europe - **SI**

May 6, 2026

Contents

A Data 2

B Additional results 9

A Data

Supplementary Figure 1: Geographical distribution of annual mean temperature. The map shows the mean value for the time period 2010-2019 using data from ERA5-Land. Dashed line indicates area of study. See Methods in the manuscript for a description of the data. Base map adapted from World Bank Official Boundaries under a Creative Commons licence CC BY 4.0.

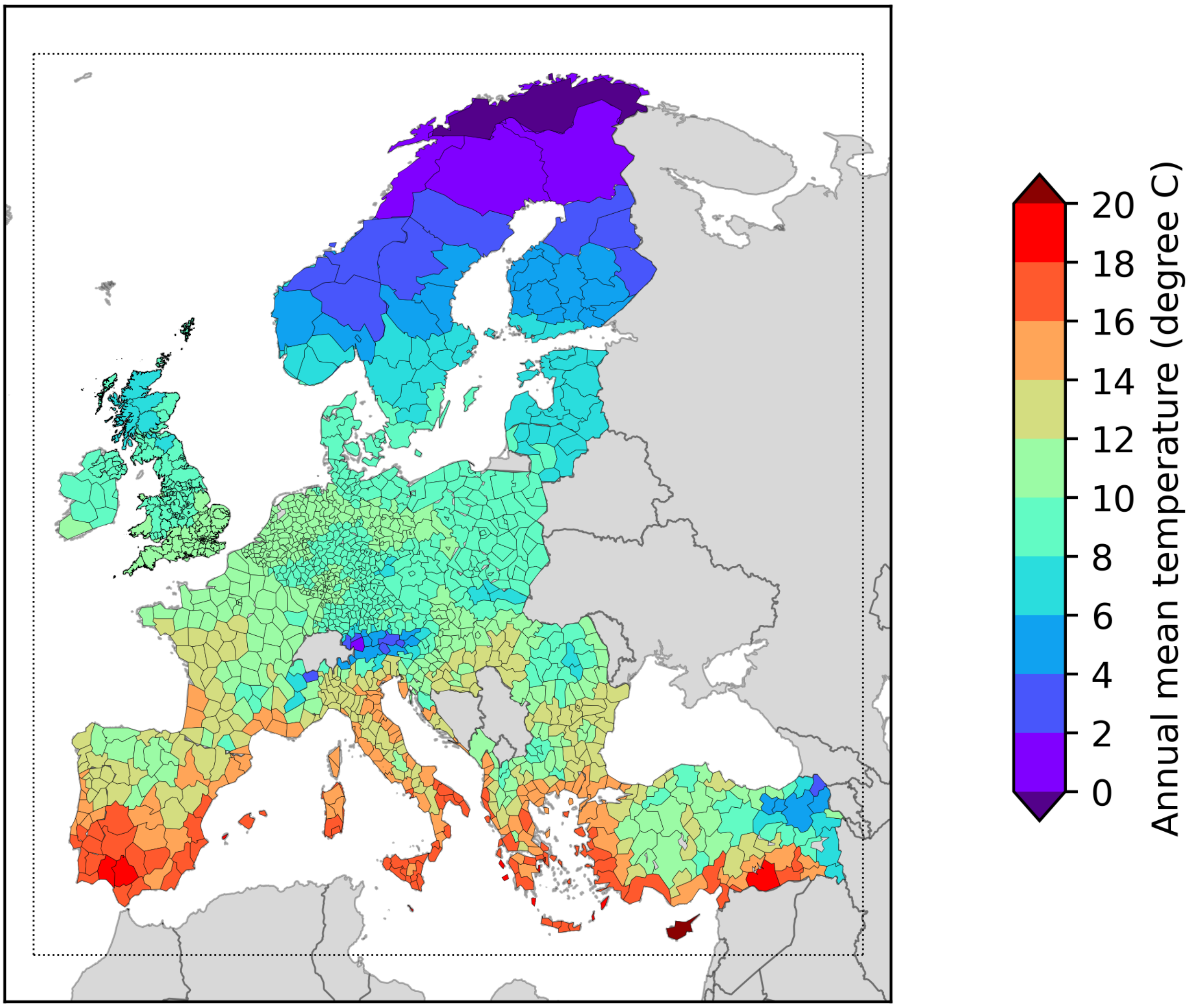

Supplementary Figure 2: Geographical distribution of GDP per capita in Europe. The map shows the mean value of the time period 2010-2019 using data from EUROSTAT. See Methods in the manuscript for a description of the data. Base map adapted from World Bank Official Boundaries under a Creative Commons licence CC BY 4.0.

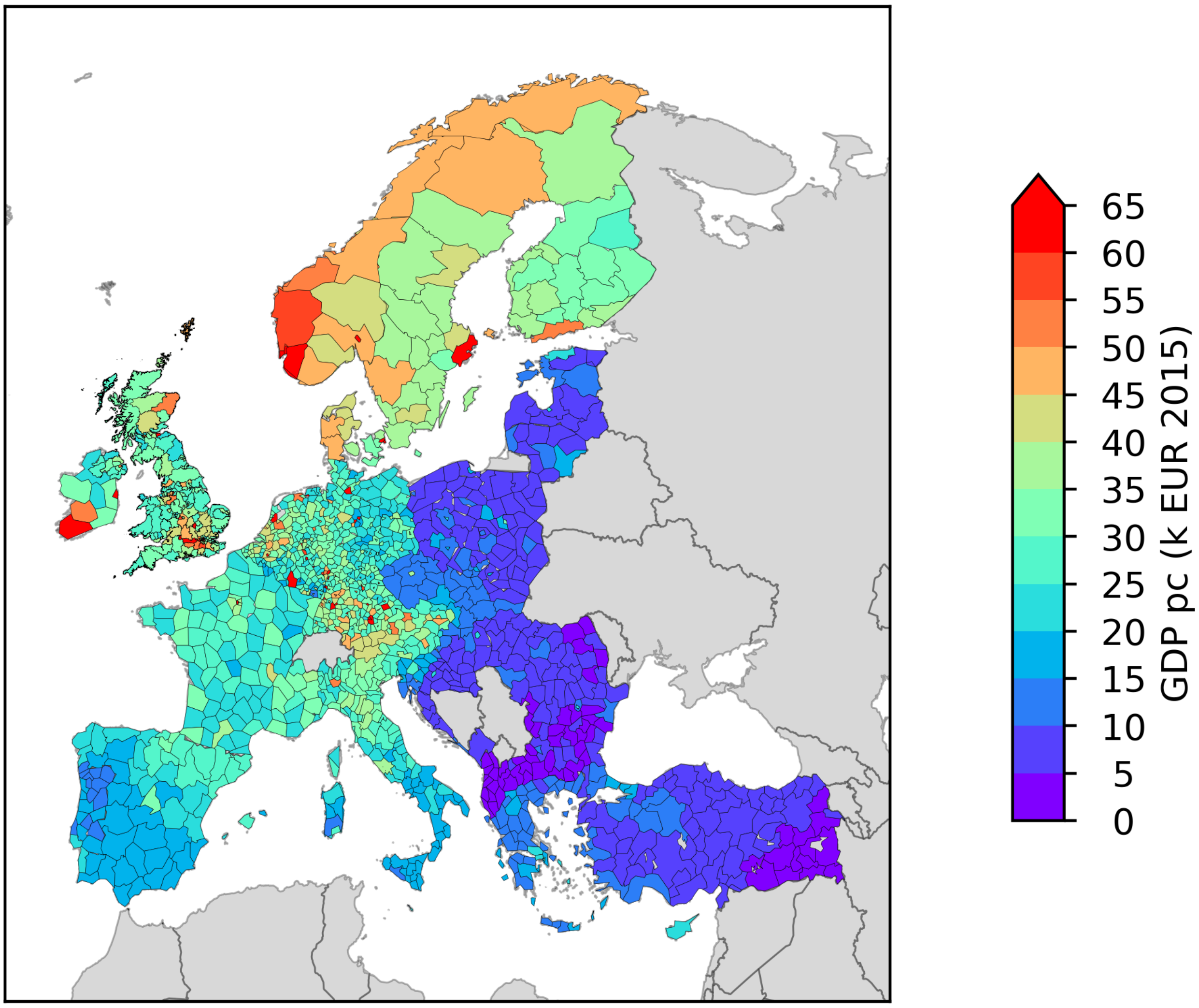

Supplementary Figure 3: Time series of GDP growth rates. Dashed vertical lines indicates the beginning of the time period that is used for the analysis. See Methods in the manuscript for explanation.

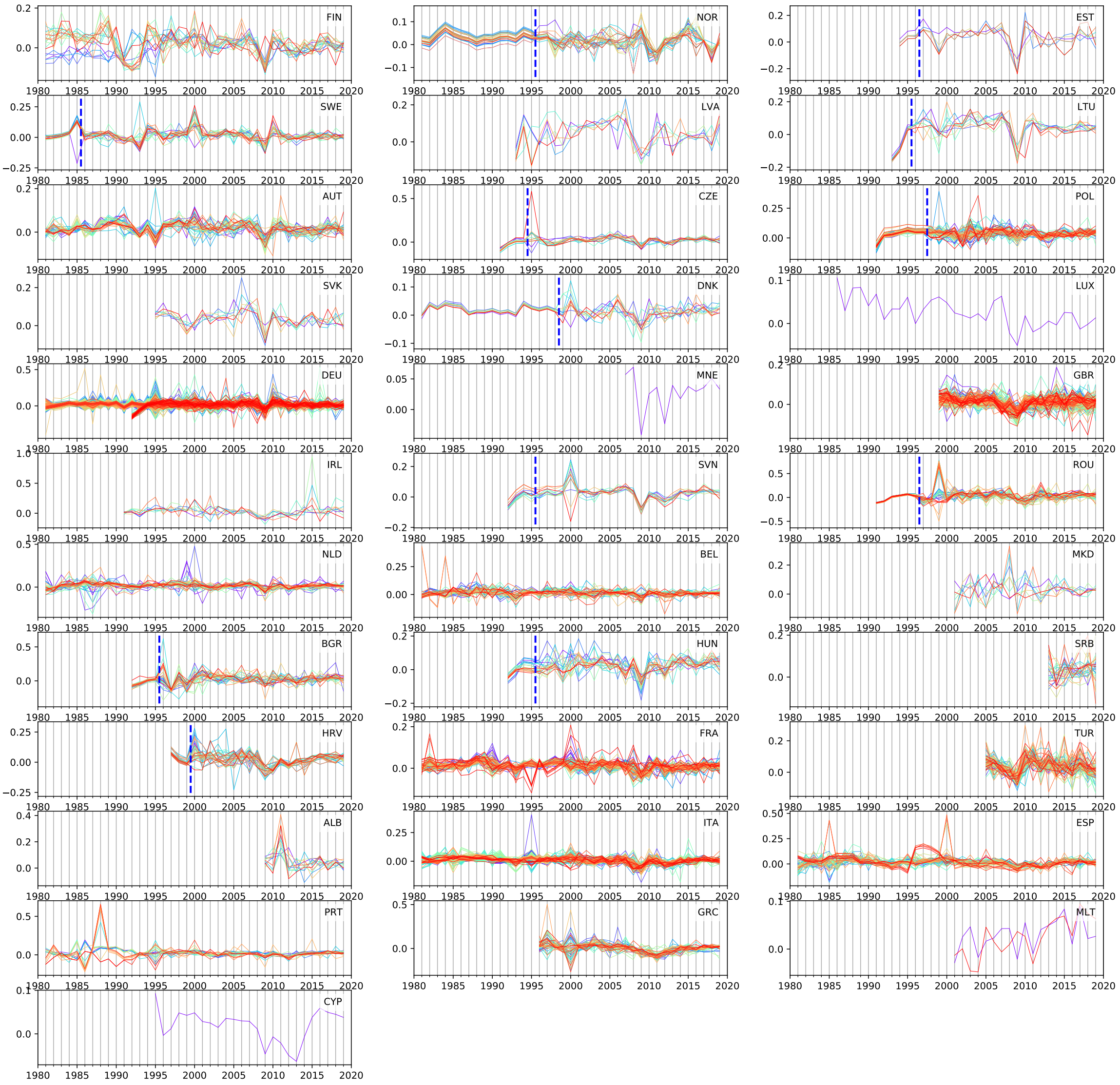

Supplementary Figure 4: Length of time series of GDP growth rates. See Methods in the manuscript for a description of the data. Base map adapted from World Bank Official Boundaries under a Creative Commons licence CC BY 4.0.

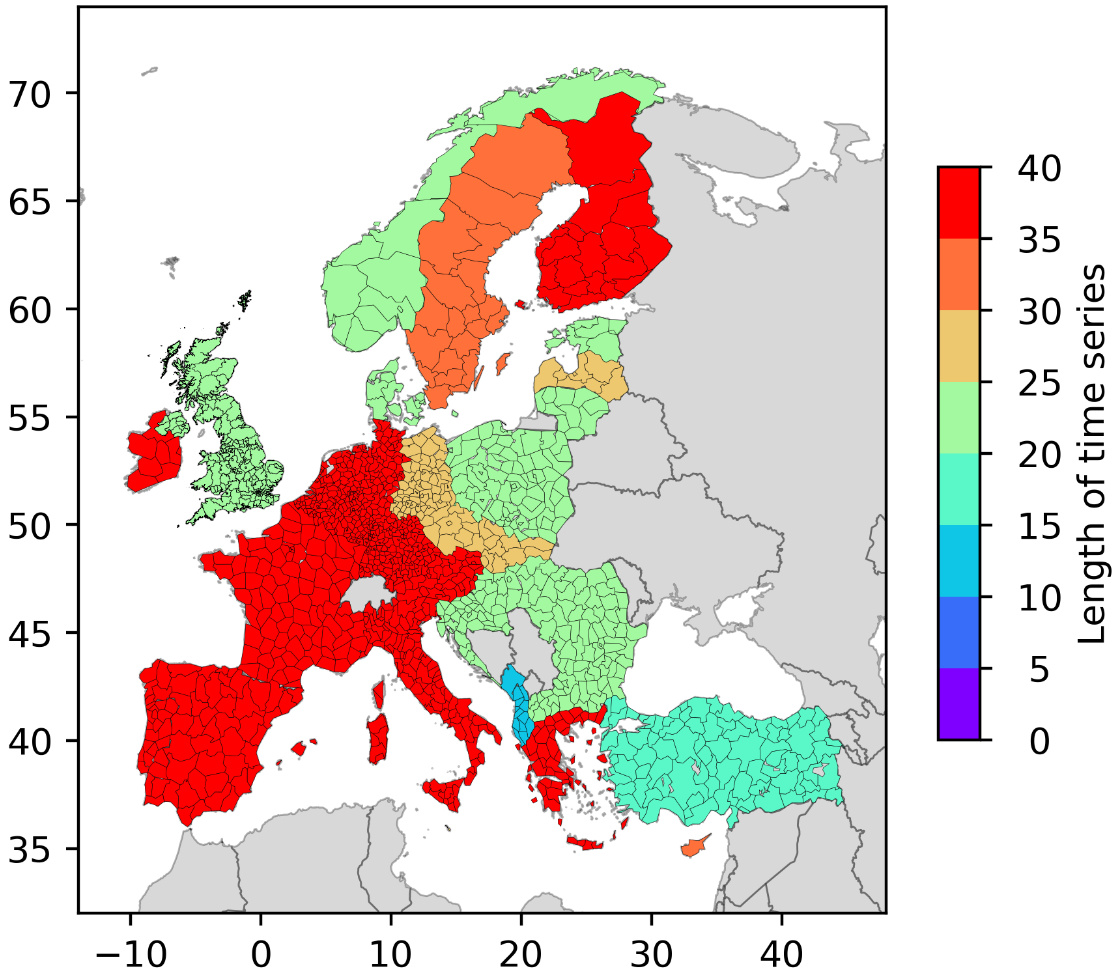

Supplementary Figure 5: Histograms of daily mean temperature of all districts of a country 1980-2019. Country ISO3 codes with the mean temperature in brackets. Continued in SI Figures 6 and 7.

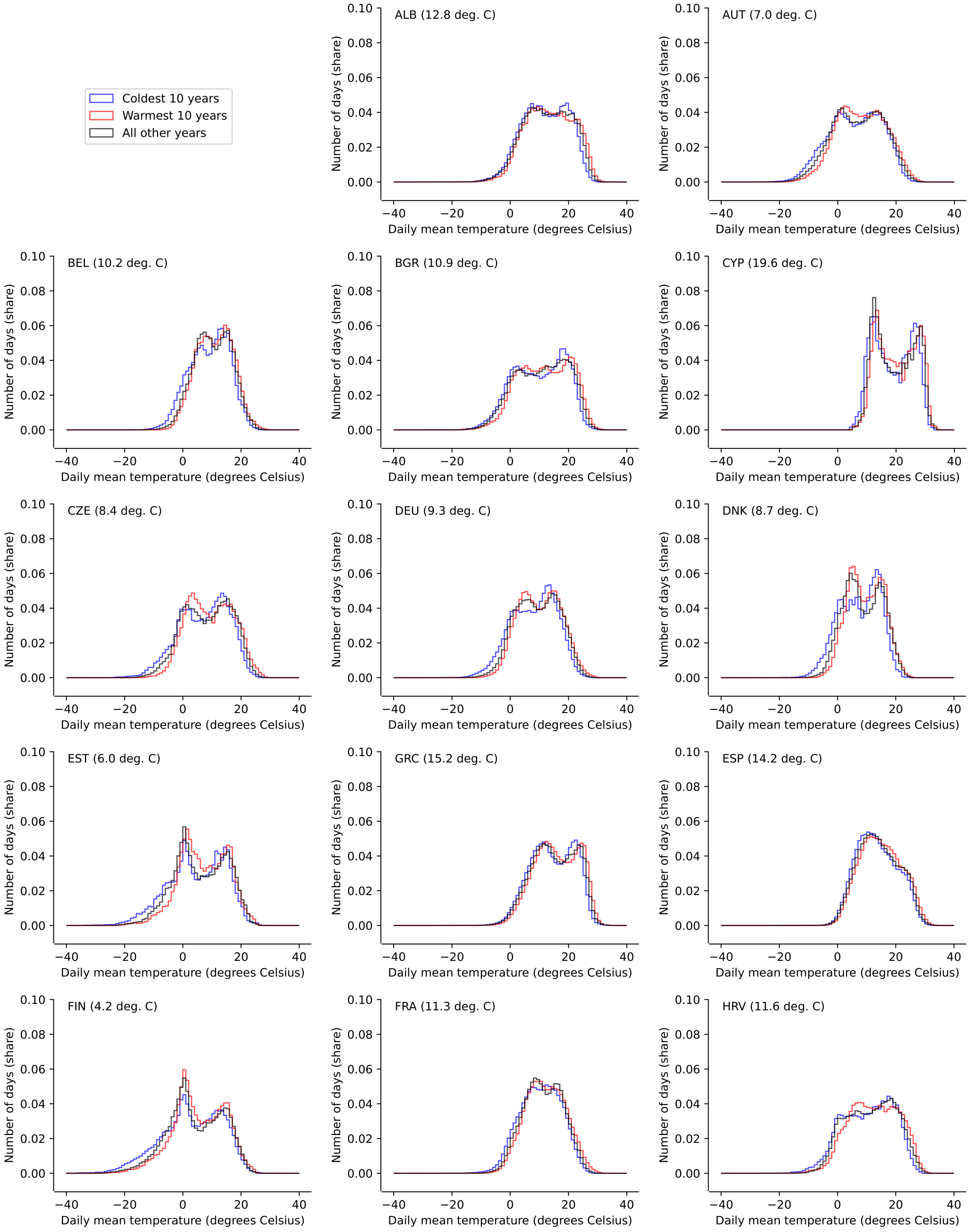

Supplementary Figure 6: Histograms of daily mean temperature of all districts of a country 1980-2019. Country ISO3 codes with the mean temperature in brackets. Continuation of SI Figure 5. Continued in SI Figure 7.

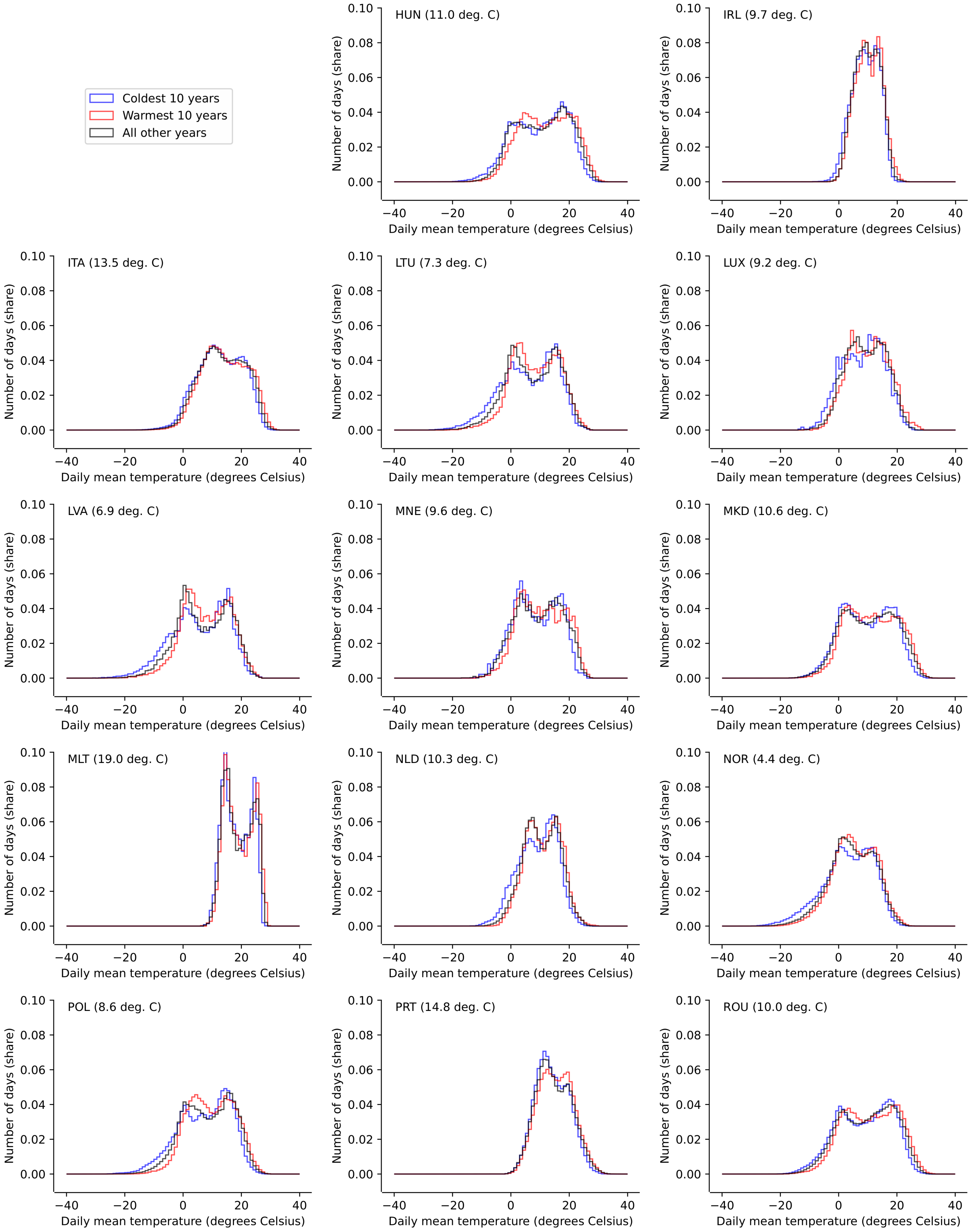

Supplementary Figure 7: Histograms of daily mean temperature of all districts of a country 1980-2019. Country ISO3 codes with the mean temperature in brackets. Continuation of SI Figure 5 and 6.

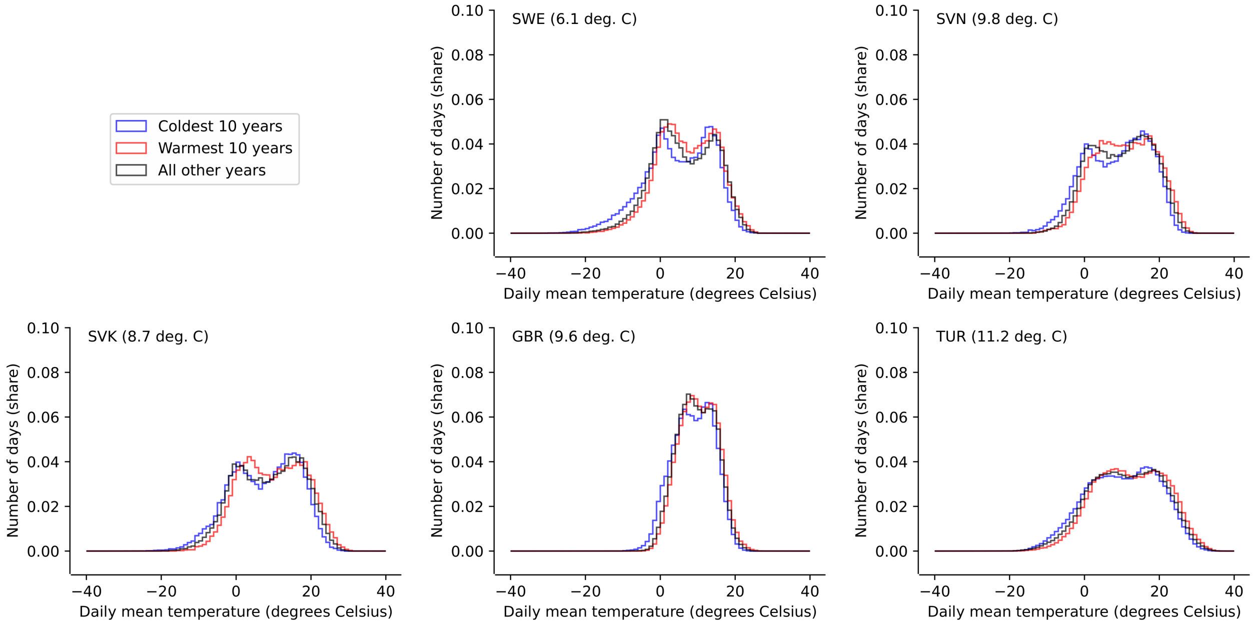

Variable	Unit	Mean	Std.	Min.	Max.	No. obs.
Population	k	372.65	497.98	18.69	15293.41	48137
GDP per capita	k 2015 EUR	24.48	18.02	1.34	543.16	48041
Annual mean temperature	deg C	10.35	2.87	-3.67	20.90	54784
Seasonal mean temperature DJF	deg C	2.47	3.82	-16.79	15.32	53592
Seasonal mean temperature JJA	deg C	18.47	3.22	7.11	31.58	54784
Annual total precipitation	m	0.00	0.00	0.00	0.01	54784

Supplementary Table 1: Descriptive statistics. The final sample includes 1494 districts and covers the years 1980-2019, with data not available for all years for all districts (see also SI Figures 4 and 3).

B Additional results

Supplementary Figure 8: Predicted and marginal effect of annual mean temperature obtained from main specification using our subnational GVA data for Europe. Shaded areas show 95% confidence intervals based on estimated standard errors.

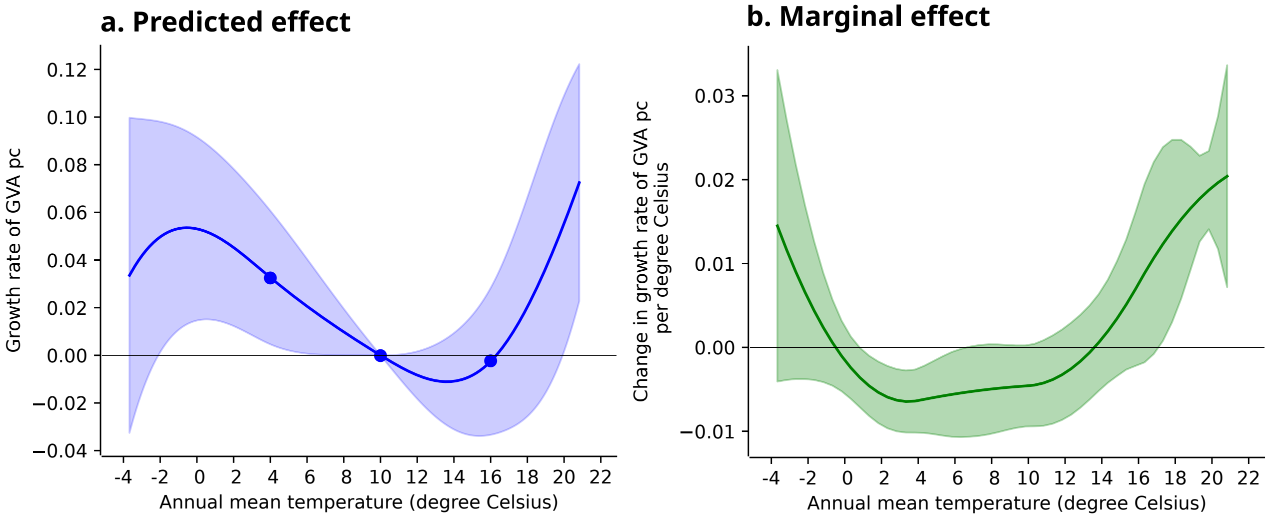

Supplementary Figure 9: Results with models with different functional forms using our subnational GVA data for Europe. Shaded areas and error bars show 95% confidence intervals based on estimated standard errors.

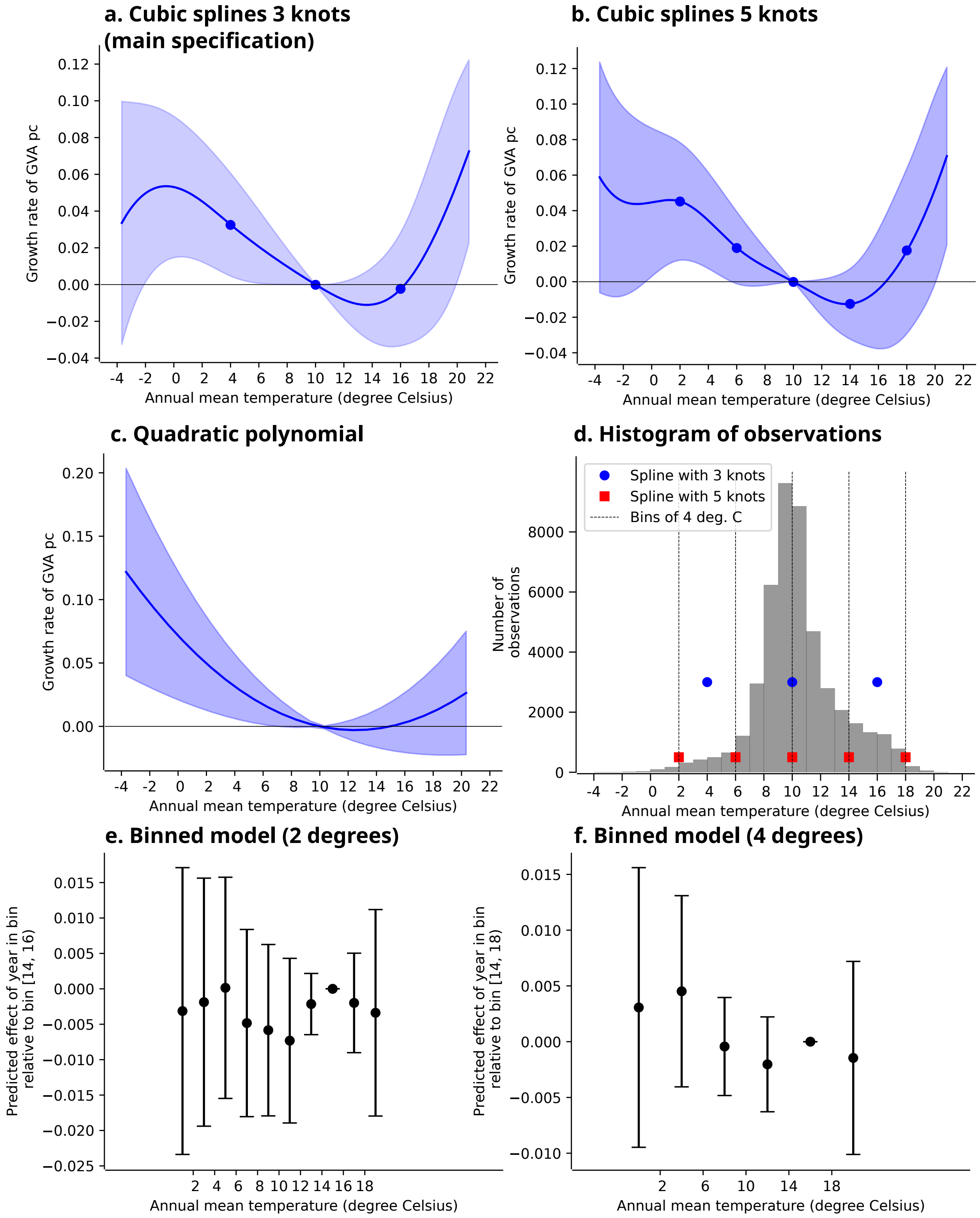

Supplementary Figure 10: Robustness tests with replication data of Burke et al. 2015 (ref. 1). Dependent variable uses GDP per capita. Shaded areas show 95% confidence intervals based on estimated standard errors.

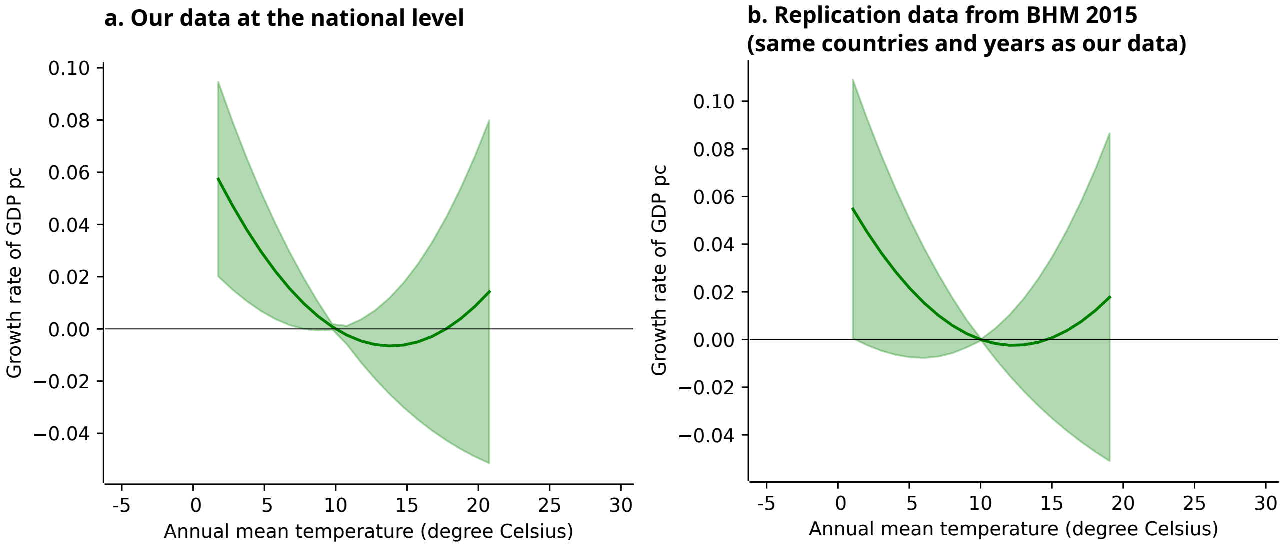

Supplementary Figure 11: Robustness tests using our subnational data for Europe: GDP instead of GVA, different time controls, and specifications with lags of GDP and temperature. Shaded areas show 95% confidence intervals based on estimated standard errors.

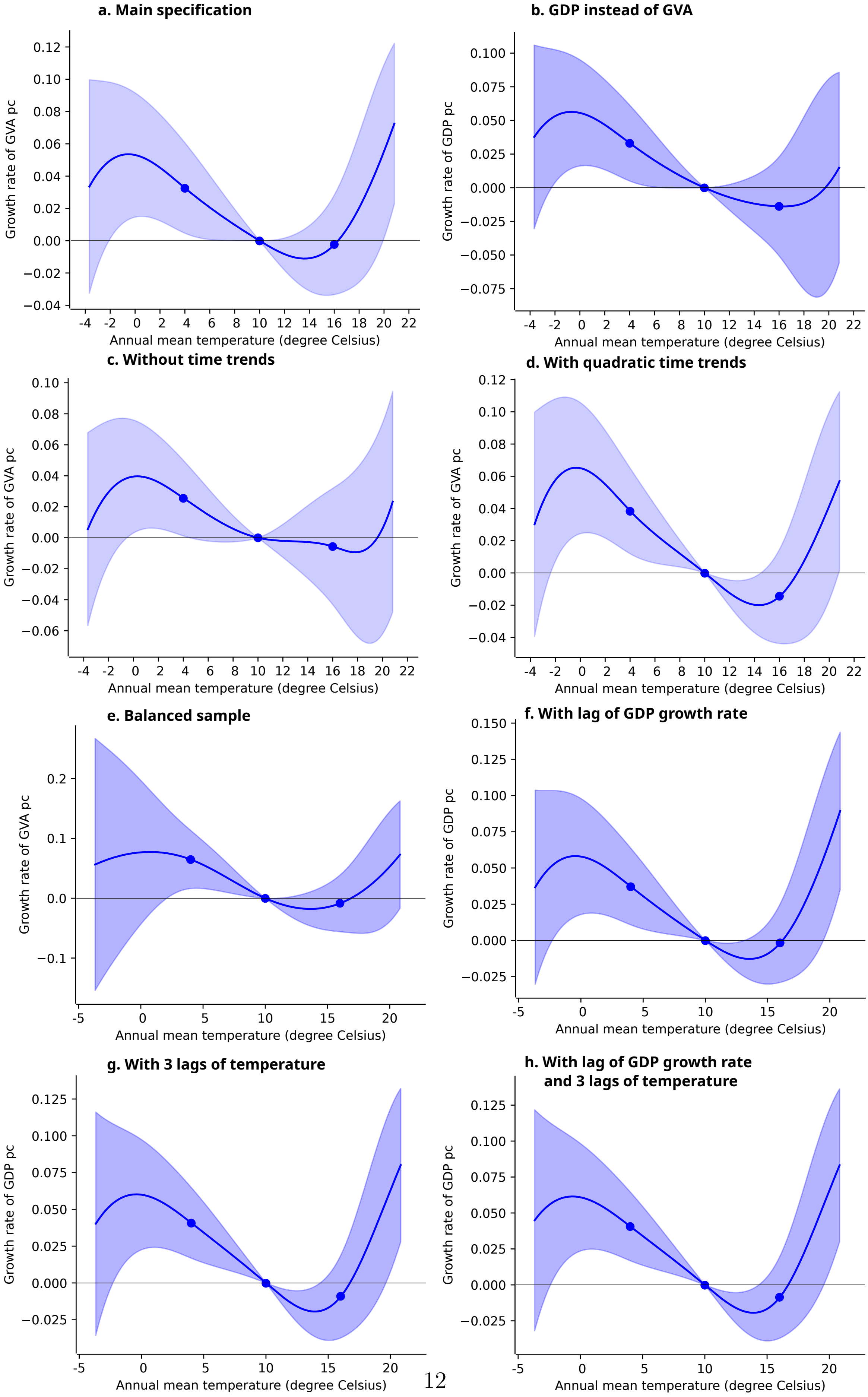

Supplementary Figure 12: Robustness tests using our subnational data for Europe: main model specification, but one model estimated for each of 36 different rainfall variables as a control variable. Each line shows the result of one model/one rainfall variable. a. Rainfall included as a linear term in the model; b. Rainfall included as a linear-by-district term in the model.

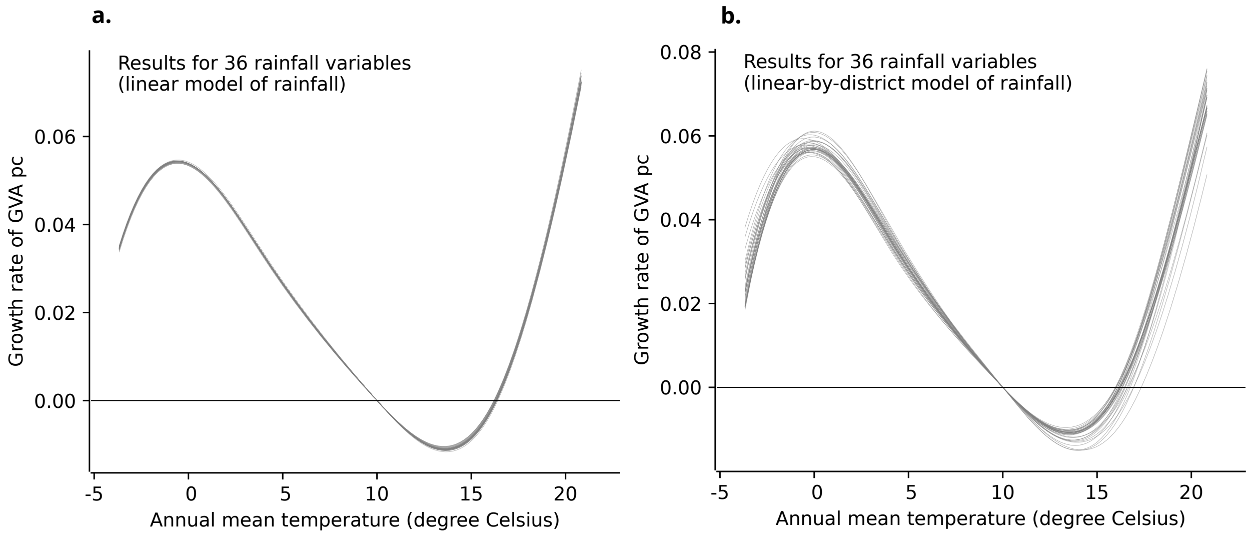

Supplementary Figure 13: Robustness tests using our subnational data for Europe: annual mean temperature interacted with dummies for different intervals of the climatological annual mean temperature of a district. We estimate this model once with our original temperature time series and once with only the "surprise" component of the time series. Error bars show 95% confidence intervals based on estimated standard errors. See Methods section for more information.

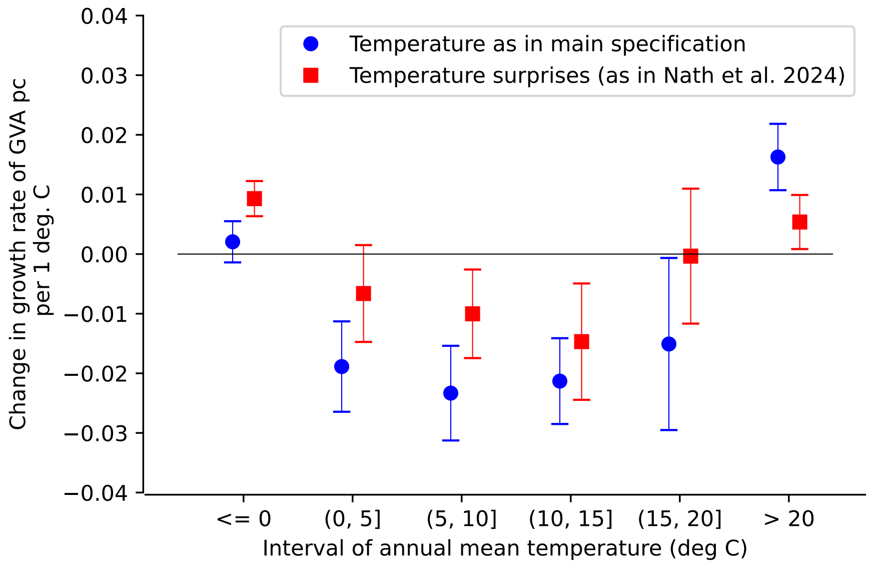

Supplementary Figure 14: Contemporaneous effects and cumulative marginal effects based on two alternative model specifications: a. quadratic model. b. linear-by-interval model. Error bars show 95% confidence intervals based on estimated standard errors. See also SI Table 2.

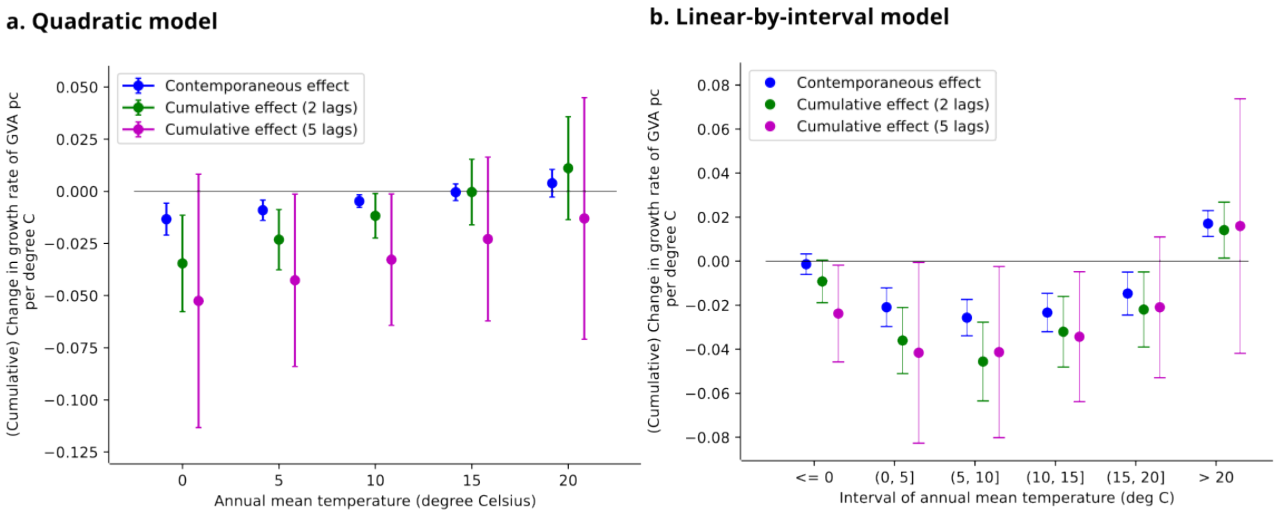

Supplementary Figure 15: Contemporaneous effects and dynamic cumulative marginal effects based on two alternative model specifications for a dynamic model (see Methods for details): a. quadratic model. b. linear-by-interval model. Error bars show 95% confidence intervals based on estimated standard errors.

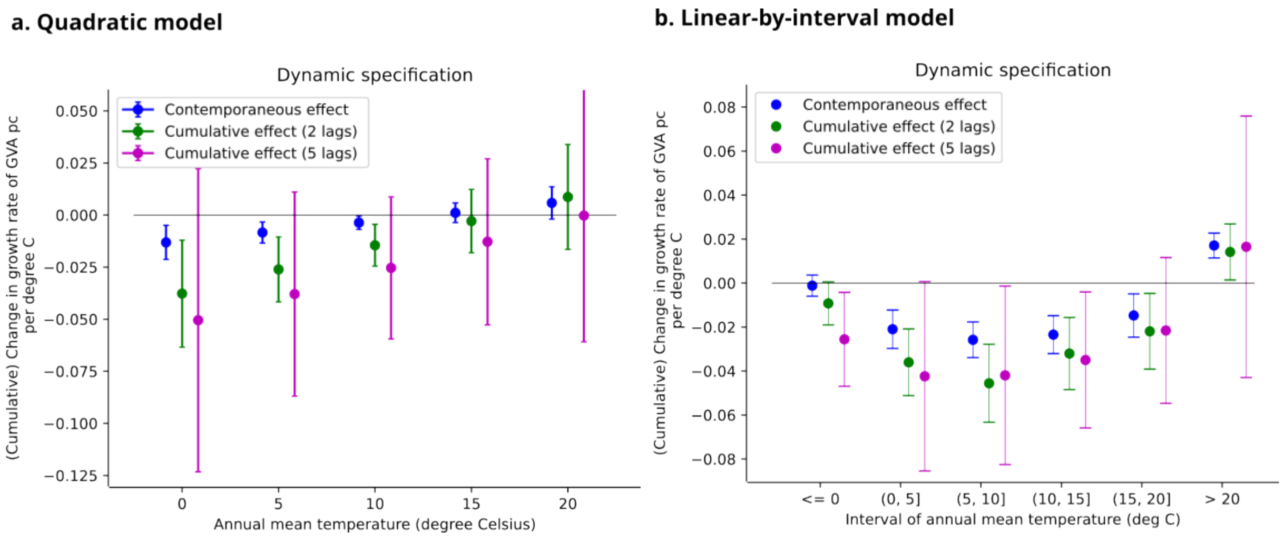

Supplementary Figure 16: Total costs for Europe for the RCP4.5 scenario (2070-2099 versus 1985-2014). a. Figure shows the median and mean estimates of the ensemble of climate models, see SI Table 3 for the results of individual climate models. The costs are quantified as the change in the average annual growth rate and expressed in percentage points. See also Figure 2a in manuscript. b. Figure shows the distribution of estimates from the ensemble of climate models.

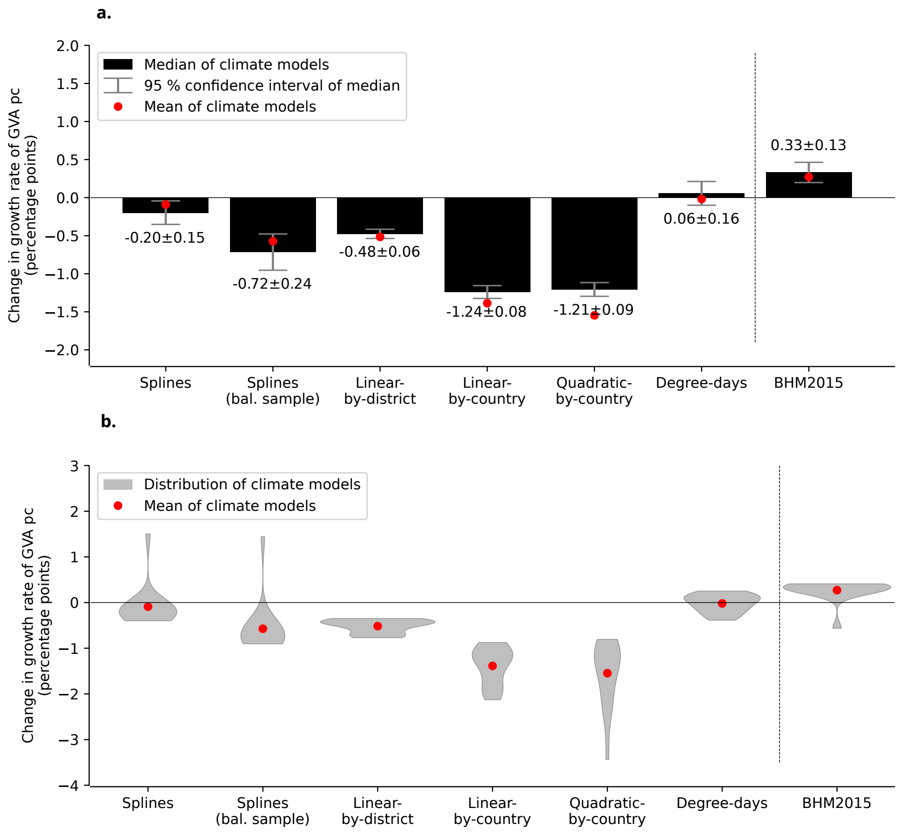

Temperature [deg C]	Number of lags		
	0	2	5
0	*** -0.013 (0.004, $<$ 0.01)	*** -0.035 (0.012, $<$ 0.01)	* -0.053 (0.031, 0.09)
5	*** -0.009 (0.002, $<$ 0.01)	*** -0.023 (0.007, $<$ 0.01)	** -0.043 (0.021, 0.04)
10	*** -0.005 (0.002, $<$ 0.01)	** -0.012 (0.005, 0.03)	** -0.033 (0.016, 0.04)
15	-0.000 (0.002, 0.84)	-0.000 (0.008, 0.97)	-0.023 (0.020, 0.25)
20	0.004 (0.003, 0.25)	0.011 (0.013, 0.38)	-0.013 (0.030, 0.66)

Supplementary Table 2: Cumulative marginal effects based on a quadratic model using our subnational GVA data for Europe. See also SI Figure 14. Central estimates with estimated standard errors and two-sided p-values in brackets (standard error, p-value). Stars indicate statistical significance: *: $p \leq 0.1$, **: $p \leq 0.05$, ***: $p \leq 0.01$.

Supplementary Figure 17: Estimated degree-day models by country (a, b) and extracted daily temperature level with the highest predicted growth rate (c, d). a. Degree-day model for Czech Republic. b. Degree-day model for Denmark. Notes: a. and b. The purple dashed line is a cubic spline fit to the estimated coefficients with weights set to the inverse of their standard errors. Error bars show 95% confidence intervals based on estimated standard errors.

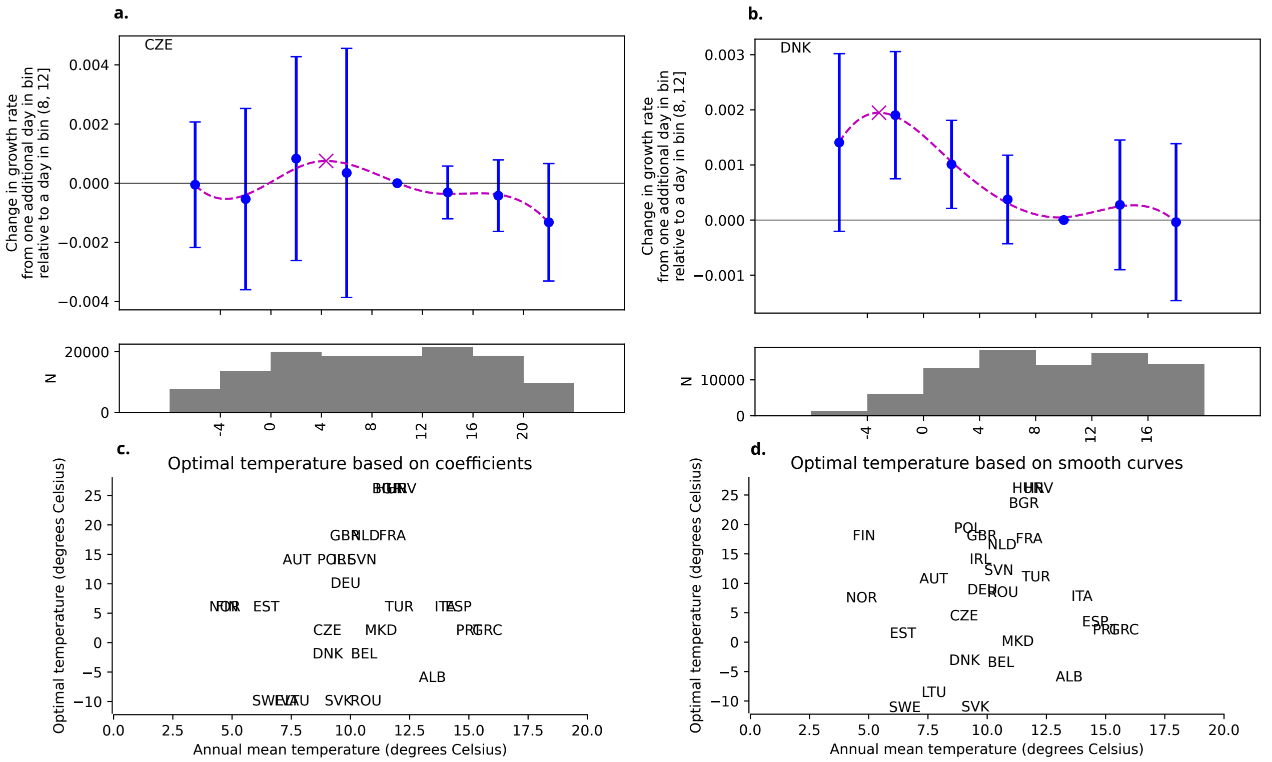

Supplementary Figure 18: Marginal effect of an increase in annual mean temperature on the growth rate of GVA in six different industry groups. **a**. Distribution of country-specific coefficients of annual mean temperature for total GVA and GVA in six industry groups over all n=32 countries in the sample. United Kingdom not shown because it uses a different sector disaggregation. Horizontal lines indicate mean values, boxes show inter-quartile ranges, and whiskers the min/max of the distributions. **b**. Country-specific coefficients of annual mean temperature for total GVA and GVA in six industry groups for individual countries. The figure is the same as Figure 4 in the main text but for a dynamic model (see Methods for details).

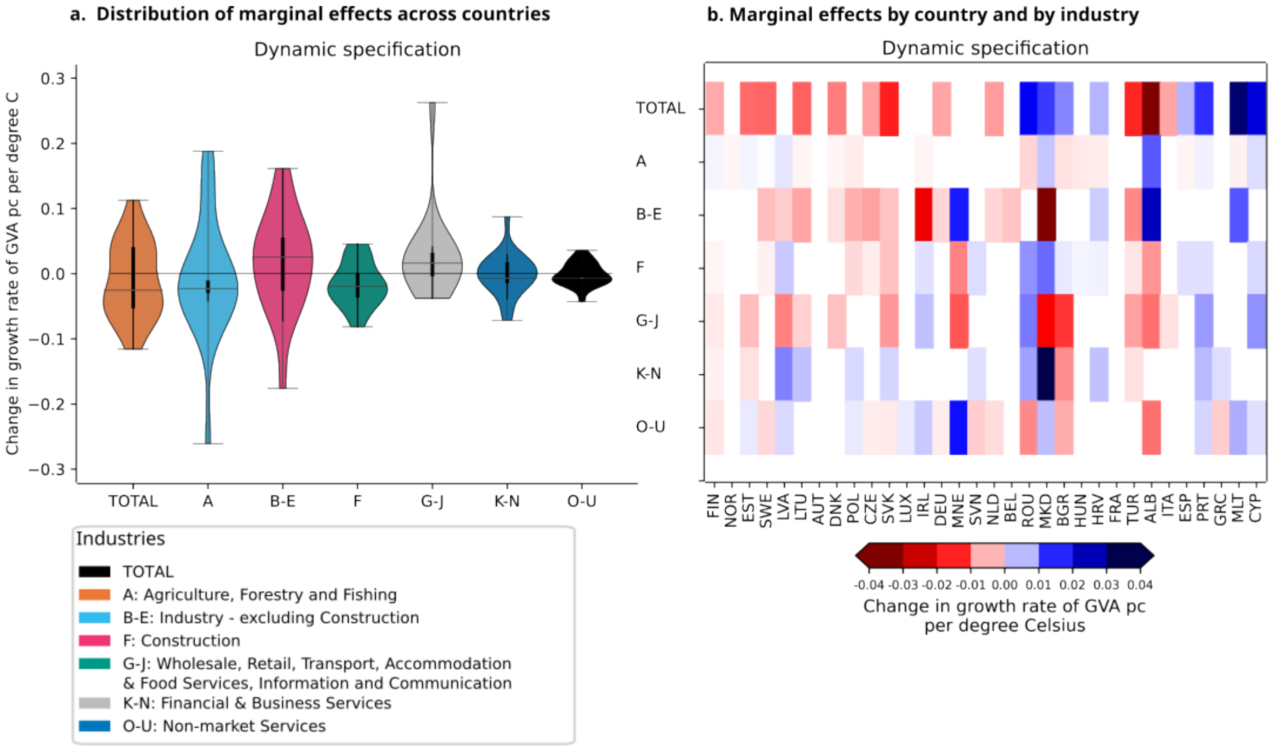

Supplementary Figure 19: Country-specific coefficients of seasonal mean temperature for total GVA and GVA in six industry groups for specific countries for winter (DJF) and summer (JJA). See manuscript Figure 4b for the effects of annual mean temperature.

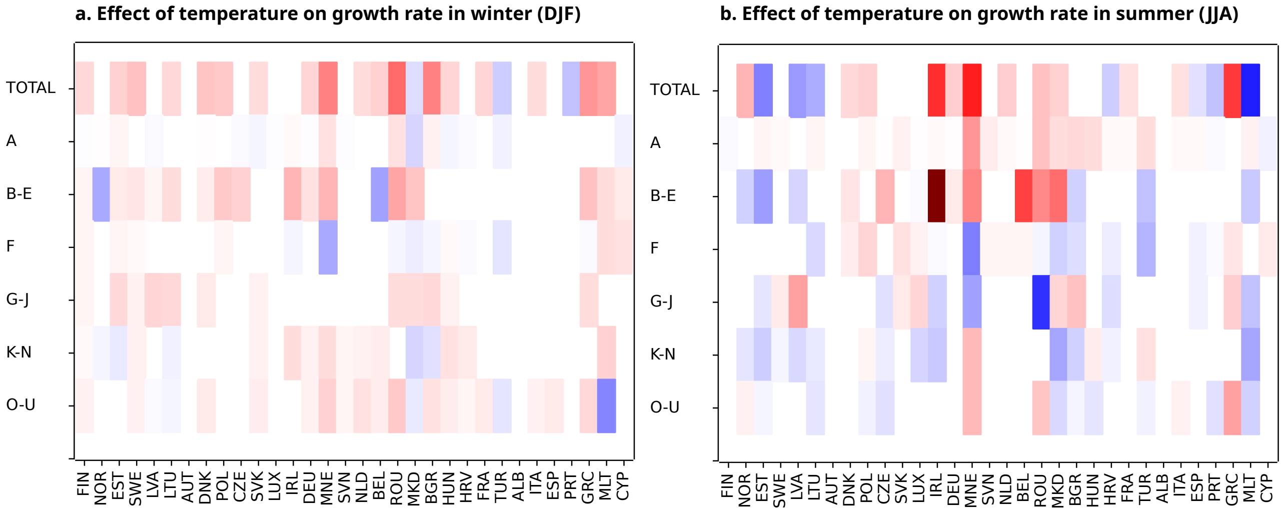

Model	Costs of future climate change in terms of the average annual growth rate (in percentage points)							
	Warming	Splines	Splines (balanced)	Linear-by-district	Linear-by-country	Quadratic-by-country	DD-by-country	BHM 2015
FGOALS-g3	1.51	-0.40	-1.11	-0.40	-1.02	-1.08	0.25	0.38
MRI-ESM2-0	1.67	-0.10	-1.02	-0.35	-0.87	-0.80	-0.39	0.23
NorESM2-LM	1.82	-0.07	-1.04	-0.35	-0.90	-0.84	-0.33	0.21
MPI-ESM1-2-HR	1.83	-0.26	-1.22	-0.39	-1.05	-1.01	-0.13	0.34
KIOST-ESM	2.03	-0.15	-1.27	-0.39	-1.17	-1.18	0.09	0.28
MPI-ESM1-2-LR	2.03	-0.24	-1.26	-0.44	-1.15	-1.20	-0.05	0.32
GFDL-ESM4	2.11	-0.30	-1.39	-0.48	-1.24	-1.21	-0.09	0.38
GISS-E2-1-G	2.25	-0.20	-1.39	-0.49	-1.24	-1.22	-0.16	0.33
MIROC6	2.33	-0.10	-1.40	-0.44	-1.21	-1.17	-0.25	0.29
BCC-CSM2-MR	2.36	-0.33	-1.48	-0.49	-1.40	-1.50	0.18	0.39
MIROC-ES2L	2.72	-0.28	-1.64	-0.61	-1.65	-1.83	0.15	0.38
ACCESS-ESM1-5	3.04	-0.05	-1.76	-0.67	-1.85	-2.08	0.06	0.30
GFDL-CM4	3.26	-0.24	-1.92	-0.75	-1.99	-2.27	0.21	0.41
CMCC-CM2-SR5	3.30	1.50	-1.30	-0.74	-1.92	-3.44	0.10	-0.56
CMCC-ESM2	3.63	-0.14	-2.08	-0.77	-2.13	-2.38	0.06	0.39
Mean	2.39	-0.09	-1.42	-0.52	-1.39	-1.55	-0.02	0.27
Median	2.25	-0.20	-1.39	-0.48	-1.24	-1.21	0.06	0.33

Supplementary Table 3: Projected costs of climate change for the RCP4.5 scenario (2070-2099 versus 1985-2014). The costs are quantified as changes in the average annual growth rate between the two periods and shown in percentage points. SI Figures 20 and 21 visualise the geographic distribution of the projected changes in temperature. Notes: DD = degree days. Splines (balanced) refers to the balanced sample that uses only the last 12 years of data that are available for all countries.

Supplementary Figure 20: Projected changes in annual mean temperature for the RCP4.5 scenario. See SI Table 3 for the average warming projected by individual climate models. Base map adapted from World Bank Official Boundaries under a Creative Commons licence CC BY 4.0.

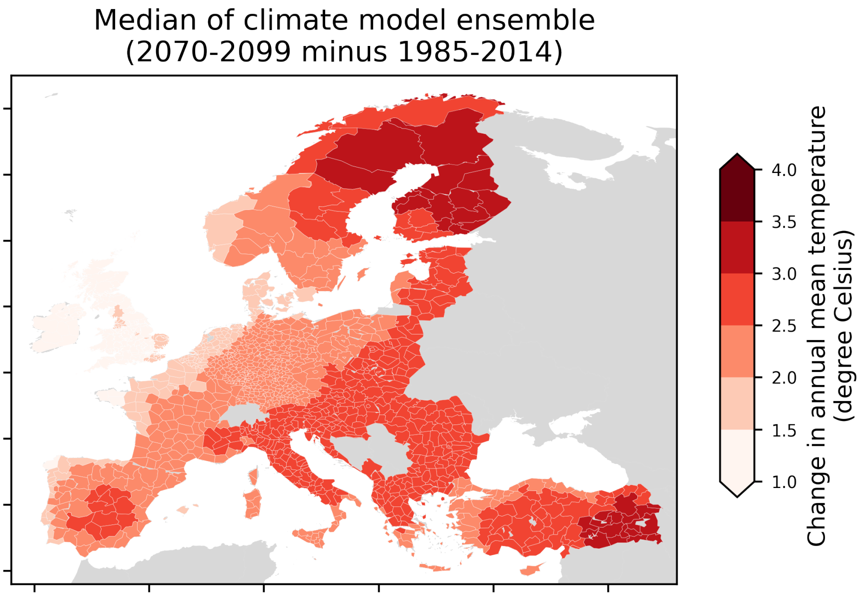

Supplementary Figure 21: Projected changes in annual mean temperature for the RCP4.5 scenario for the two "coldest" (top row) and the two "warmest" (bottom row) climate models based on the projected average warming in Europe. Note the different scales. See SI Table 3 for the average warming projected by individual climate models. Base map adapted from World Bank Official Boundaries under a Creative Commons licence CC BY 4.0.

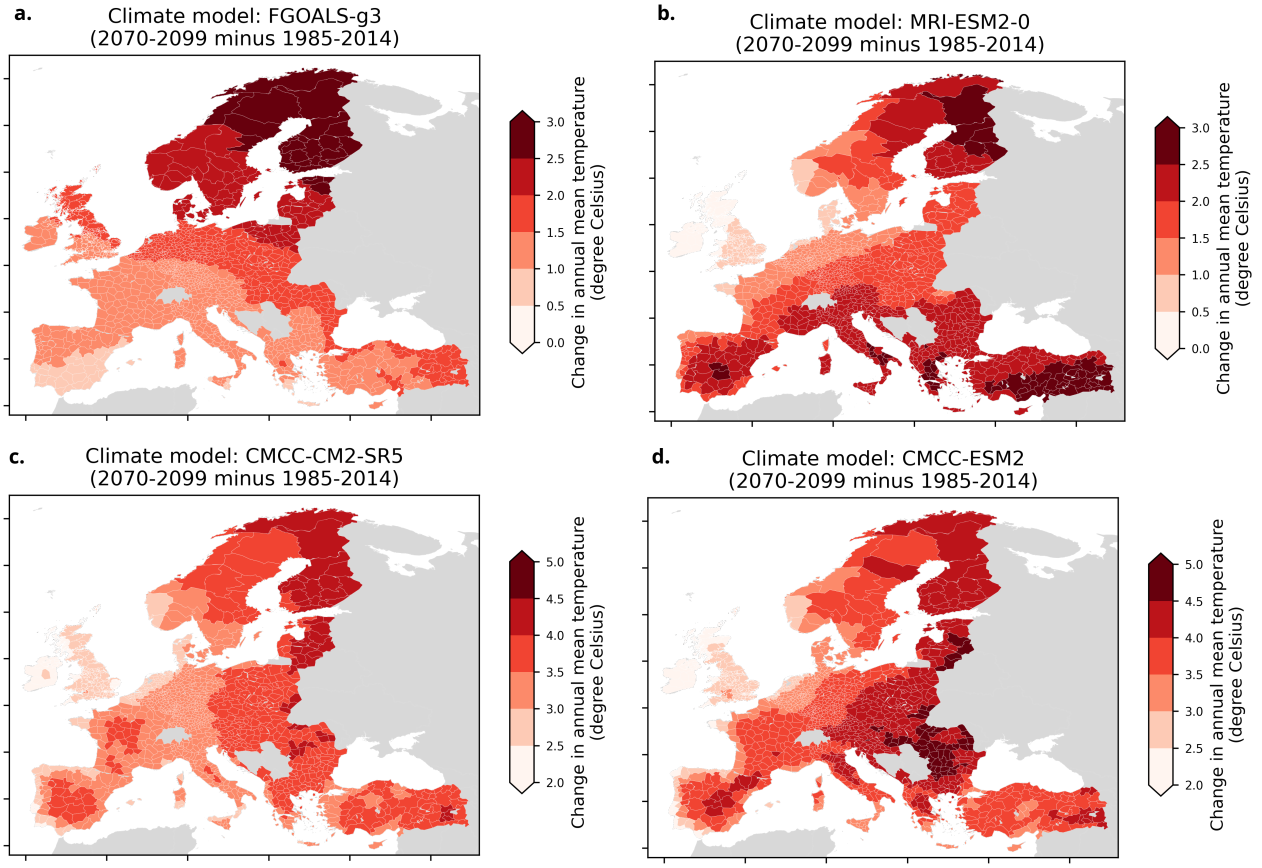

Supplement: Supplementary file 1 — Supplementary Information [file 41467_2026_73341_MOESM1_ESM.pdf]
