# Supplementary material for: Economic specialization and heterogeneous temperature-economy relationships suggest net costs of climate change in Europe
Source: Nat Commun. 2026 Jun 5;17:7194. doi: 10.1038/s41467-026-73341-4 (PMC13396729; doi:10.1038/s41467-026-73341-4)
Supplement: Supplementary file 2 — Peer Review file [file 41467_2026_73341_MOESM2_ESM.pdf]

nature portfolio

Peer Review File

Economic specialization and heterogeneous temperature-economy relationships suggest net costs of climate change in Europe

Corresponding Author: Professor Ben Groom

This file contains all reviewer reports in order by version, followed by all author rebuttals in order by version.

Version 0:

Reviewer comments:

Reviewer #1

(Remarks to the Author)
The paper is very comprehensive using numerous different definitions of variables and econometric specifications to demonstrate the robustness of the results. Overall, the evidence is convincing.

The main area that I believe could be improved in the paper at relatively low cost to the authors is to explicitly explore the role of temperature fluctuations in different seasons. This could help to reconcile the results with previous results or more clearly illustrate the mechanisms behind the differences. Particularly, paired with sectoral level economic data, seasonal temperature data could help to understand the heterogeneity across geographic areas. The paper contains a short description of seasonal temperatures with regards to fluctuations in annual mean temperatures and it includes an analysis using degree days but degree days disregard the timing of these days. For instance, in Europe, a 20C day in July is likely to have very different impacts than a 20C day in January. Seasonal temperatures could help to bridge the gap.

(Remarks on code availability)

Reviewer #2

(Remarks to the Author)

(Remarks on code availability)
Referee report for Nature Communications
Some like it cold!: Heterogeneity in the temperature-economy relationships of Europe

Summary

The paper examines the relationship between temperature and economic activity over the period 1980-2019, using NUTS3 (i.e. provincial) data for Europe. The temperature-growth relationship is analyzed through fixed-effect panel regression techniques, where the growth rate of annual gross value added (GVA) per capita is function of annual temperature (and precipitation). While the empirical literature typically models this relationship through a quadratic-in-temperature specification (as in Burke et al., 2015), the authors use spline regressions, which do not impose a predetermined polynomials to model non-linearities. Unlike most studies that find an inverted U-shape relationship for global economies, the results for Europe show a U-shape relationship between temperature and economic growth for initial temperatures between 0°C and 20°C degrees. Specifically, cold (hot) regions show a negative (positive) effect of temperature rise on GVA per capita growth. There is evidence of a beneficial (detrimental) impact of temperature shocks only at extreme temperatures, i.e. below 0°C degrees (above 20°C degrees). The authors also investigate potential transmission channels through which temperature affects economic growth, finding significant heterogeneity across provinces (both within and between countries) and economic sectors.

General comments

The topic of the paper is highly relevant and fits well within the growing literature on the temperature-growth nexus. The findings are particularly interesting, as they offer new insights into the complexity of the relationship between temperature and economic activity, moving beyond the traditional hot vs. cold region comparison. I like the idea of not relying on predetermined polynomials to model the non-linear relationship between temperature and economic activity (e.g., Burke et al., 2015, use a quadratic polynomial, Newell et al., 2021, rely on higher-order polynomials). The authors conduct rigorous robustness checks, showing that their results are not driven by data selection or model specification. Moreover, they provide compelling explanations for their findings, emphasizing the role of sectoral differences in shaping the temperature-growth relationship. However, there are a few areas I would recommend the authors to further explore.

Comments

Contribution

1. The Introduction is well-written and clear, but I would suggest emphasizing more explicitly the main contribution of the paper. Is the main innovation the empirical approach (i.e., the use of spline regressions), or does it lie in the use of more granular data at the NUTS3 level? As for the latter, a recent ECB working paper (i.e., Usman et al., 2024) also examines the temperature-growth relationship using NUTS3 data for Europe. I suggest comparing your results with those of Usman et al. (2024).

Empirical analysis

2. It is not clear whether the f(.) functional is linear when the authors turn their focus on slope heterogeneity, by allowing temperature effect on growth to differ by district or by country (see pag. 8 in the econometrics frameworks section).

3. It would be helpful for the reader to use, in the econometric framework, footnote in table s3 to replace the description of how the authors compute projections. Moreover, in the discussion of results, it would be helpful to describe how do these projections compare with findings in the literature for global economies (see for instance the recent paper by Bilal and Känzig (2024)

4. The authors estimate a static panel regression with no lagged temperature levels as additional regressors. However, the recent study by Nath et al. (2024) points at the importance of including lags of temperatures (and GDP) in the model specification, given the serial correlation typically observed in these series. Do the main results remain similar when lags of temperature and GDP are included?

5. Several studies have shown that temperature shocks affect economic activity through changes in labour productivity (see, e.g., Acevedo et al., 2020; Mohaddes et al., 2024, among others). What role does labour productivity play in your analysis? Furthermore, it would be interesting to explore also how investments in different sectors respond to rising temperatures.

6. Why do the authors use a quadratic polynomial for precipitations, while they use cubic splines for temperature? Is this choice motivated by evidence in the empirical literature?

7. The authors should define, in the Conclusions, the limitation of their study (for instance, labor productivity and investments are transmission channels which have not been explored) suggesting future scope of research

Minor comments

1. I suggest adding notes below the figures and tables reported in the Appendix.

References

- Acevedo, S., Mrkaic, M., Novta, N., Pugacheva, E., & Topalova, P. (2020). The Effects of Weather Shocks on Economic Activity: What are the Channels of Impact? Journal of Macroeconomics, 65, 103207.
- Burke, M., Hsiang, S. M., & Miguel, E. (2015). Global non-linear effect of temperature on economic production. Nature, 527, 235-239.
- Bilal, A., & Känzig, D. R. (2024). The Macroeconomic Impact of Climate Change: Global vs. Local Temperature. NBER Working Paper No. 32450, National Bureau of Economic Research.
- Mohaddes, K., Ng, R. N. C., Pesaran, M. H., Raissi, M., & Yang, J.-C. (2023). Climate change and economic activity: evidence from US states. Oxford Open Economics, 2, odac010.
- Nath, I. B., Ramey, V. A., & Klenow, P. J. (2024). How Much Will Global Warming Cool Global Growth? NBER Working Paper No. 32761, National Bureau of Economic Research.
- Newell, R. G., Prest, B. C., & Sexton, S. E. (2021). The GDP-Temperature relationship: Implications for climate change damages. Journal of Environmental Economics and Management, 108, 102445.
- Usman, S., Fernandez, G. G. T., & Parker, M. (2024). Going NUTS: the regional impact of extreme climate events over the medium term. ECB Working Paper Series No. 3002, European Central Bank.

Version 1:

Reviewer comments:

Reviewer #1

(Remarks to the Author)
The authors have addressed the reviewers' primary comments.

The results are still somewhat counter-intuitive based on some of the existing literature. In particular, it would be helpful for the authors to compare their findings to "Seasonal Temperatures and Economic Growth in the United Kingdom" by Lotanna E. Emediegwu, Veronica Vienne, and Jubril Animashaun in the Oxford Bulletin of Economics and Statistics, which find different seasonal patterns of effects in the UK.

Can you explain why your results differ?

(Remarks on code availability)

Reviewer #2

(Remarks to the Author)
Referee report for the manuscript: Some like it cold!: Heterogeneity in the temperature-economy relationships of Europe, ref. NCOMMS-24-68298A

The authors carried an extensive revision of the manuscript addressing some of the issues raised. The following are my comments:

1) As for the issue of temperature seasonality, it would be useful to clarify which months are included in winter and which one in summer? It would be interesting to know whether the authors aggregate six months for each season, since, in this case, they won't be able to capture further observations concentrated in the distribution tail which, I guess, might corroborate the evidence of non-linearities in temperature-growth relationship discovered for Europe.

2) As for the specification with lags in the dependent variable and in temperature, the authors should fully account the Nath et al (2024) insights:
a) if there is any evidence serial correlation in temperature, then it would be worthwhile to filter out the anticipated component of temperature and identify the shock to temperature and to explore its effect on growth.
b) more importantly, the focus on panel (with long time series) allows to draw conclusion on growth vs level effect (Nath et al, emphasize persistence effects as an intermediate case). In other words, the authors, in the revised draft, control for lags in temperature and growth (dependent variable) but they only report the impact coefficients (there might be evidence, in line with other panel studies, of sign reversals over medium term horizons). Therefore, it would be useful to know whether the non-linear model specification holds for horizon beyond one year.

3) The slope heterogeneity (across districts) is controlled trough panel linear regression with the slope coefficients varying across districts. It is not clear whether, in this case, the authors estimate by OLS a time series regression for each district or if they pool the data and include fixed (cross sectional, time fixed effect, and heterogeneity in the slope coefficients). The second model has a proliferation of parameters which might require to resort to some shrinkage estimation methods. The first specification can be recast within the Mean Group approach and it can be handled by OLS and then the authors can average across different groups (districts with temperature below 0 Celsius degree, etc...)

References

- Nath, I. B., Ramey, V. A., & Klenow, P. J. (2024). How Much Will Global Warming Cool Global Growth? NBER Working Paper No. 32761, National Bureau of Economic Research.

(Remarks on code availability)

Version 2:

Reviewer comments:

Reviewer #1

(Remarks to the Author)
The authors have addressed the reviewers' concerns by estimating additional models. There is the potential for future research to expand on this paper but that is beyond the scope of this paper.

(Remarks on code availability)

Reviewer #2

(Remarks to the Author)
The authors have addressed most of the issues I raised. As for my comments B2 b) I have the following remarks.

The authors stress that their contribution is with respect to Burke et al (2015) and they add that they do not think it is helpful to add additional methodological novelties that have not yet been published in a peer-reviewed journal.
However, I argue, the literature on a (non-linear) relationship between temperature and output has since then produced several peer-reviewed journal publications which focus not only on contemporaneous linkages, but on dynamic linkages and on the level vs growth effect of temperature. As I commented in my previous referee report, the NBER working paper (which is a prestigious publication, subject to peer review) by Nath et al, contributes to the debate growth vs level effect, taking into account persistence in both temperature and output. My suggestion, then, is to include full dynamics and, in particular, to explore not only a contemporaneous but also a relationship beyond one year horizon. Although the authors follow my suggestion (see B2 a of my previous report) and they estimate temperature shocks, I guess that the model specification producing the results in Fig 6 in the supplementary material and also in the rebuttal letter is static (e.g. the authors explore only the contemporaneous impact of a temperature shock on growth).
Therefore:

1) Can the authors provide an equation, at least, in the supplementary material describing the model specification underlying Fig 6?

In general, to my understanding, all the figures produced in the main text and in the supplementary material are on the contemporaneous (one year) relationship between output and temperature (or temperature shocks). This should be explicitly stated in the main text. The only exceptions are in the rebuttal letter which shows a Figure on "Cumulative Change in growth rate of GVA pc per degree Celsius". These figures have with two panels: the right hand side named "Dynamic Specification" and the left hand side which, I guess, it is based on a non-dynamic specification which, however, is the only one showing up in the supplementary materials (see Fig B7 in SI). Therefore:

2) Can the authors provide an explanatory equation (and the associated code to reproduce the results) describing the Figures in the aforementioned two panels and, in particular, the one for the dynamic specification (which is the one I asked for in my previous report)? I could not find such explanatory equation in the main text nor in the supplementary material. Moreover, the codes needs a README file to see the different model specifications including those producing Figures B6 and B7 (especially how you compute the compute the cumulative marginal effects in Fig B7, SI).
As long as the results in Figure B7, SI, are associated with the outcome of a dynamic panel regression (please specify an equation describing the model and how you compute the cumulative marginal effects), the empirical findings suggest that only in cold countries there is evidence of a statistically significant negative effect of temperature for an horizon up to two years ahead (a five year horizon implies statistically insignificant empirical findings regardless of temperature levels).
In particular, in Fig B7 (SI) the authors use a dynamic model specification which is a quadratic polynomial. Therefore:

3) Is there any particular reasons why the authors do not extend the dynamic model specification also to cubic splines (they focus only on the quadratic model of Burke et al (2015)) since it is the main model specification producing Fig B1,B2,B4 and B5 in the supplementary material beyond Fig 1a in the main text?

4) Can the authors extend the dynamic model specification also to further robustness checks such as those for marginal effects by industry and by country (Fig 4 in the main text)?

(Remarks on code availability)
As apecified in the referee report, it would be more more helpful to have a README file

Version 3:

Reviewer comments:

Reviewer #2

(Remarks to the Author)
The authors addressed all the issues I raised in an exhaustive and convincing way
Therefore I reccomend tò accept the manuscript

(Remarks on code availability)
I am happy with the code content

Open Access This Peer Review File is licensed under a Creative Commons Attribution 4.0 International License, which permits use, sharing, adaptation, distribution and reproduction in any medium or format, as long as you give appropriate credit to the original author(s) and the source, provide a link to the Creative Commons license, and indicate if changes were made.
In cases where reviewers are anonymous, credit should be given to 'Anonymous Referee' and the source.
The images or other third party material in this Peer Review File are included in the article’s Creative Commons license, unless indicated otherwise in a credit line to the material. If material is not included in the article’s Creative Commons license and your intended use is not permitted by statutory regulation or exceeds the permitted use, you will need to obtain permission directly from the copyright holder.
To view a copy of this license, visit https://creativecommons.org/licenses/by/4.0/

Responses to reviewers

Original comments in black, responses in **blue**.

Reviewer 1

The paper is very comprehensive using numerous different definitions of variables and econometric specifications to demonstrate the robustness of the results. Overall, the evidence is convincing.

A1) The main area that I believe could be improved in the paper at relatively low cost to the authors is to explicitly explore the role of temperature fluctuations in different seasons. This could help to reconcile the results with previous results or more clearly illustrate the mechanisms behind the differences. Particularly, paired with sectoral level economic data, seasonal temperature data could help to understand the heterogeneity across geographic areas. The paper contains a short description of seasonal temperatures with regards to fluctuations in annual mean temperatures and it includes an analysis using degree days but degree days disregard the timing of these days. For instance, in Europe, a 20C day in July is likely to have very different impacts than a 20C day in January. Seasonal temperatures could help to bridge the gap.

RESPONSE:

Thank you for your positive response to the paper and for this great suggestion. We have undertaken a detailed analysis of the seasonality described in your comment and summarised in two detailed figures that we now place in Section 2 of the supplementary information. These figures (SI Figures B8 a,b) complement Figure 4 in the main text. We have referenced these figures and the seasonal aspects of the heterogeneous effects in the discussion around Figure 4 in the main text.

In particular, at the end of paragraph 2 on page 6 we have added the following text:

> "We add further texture to the heterogeneous effects across sectors and countries by splitting the estimation shown in Figure 4b into responses to temperature shocks in summer (JJA) and winter (DJF) (SI Figure B8). This analysis shows that the impact of temperature shocks is rather consistent throughout the year in most sectors, with some moderate seasonality in Trade (G-J). The results also suggest that, overall, it is especially warmer-than-average winters that are harmful for economic activity, with less consistent results for warmer-than-average summers. This pattern is particularly pronounced in the coldest countries. "

SI Figure B8 is made up of the following two exhibits which mirror the presentation in Figure 4b in the main text. We think that this is a good way to display the seasonal differences and similarities.

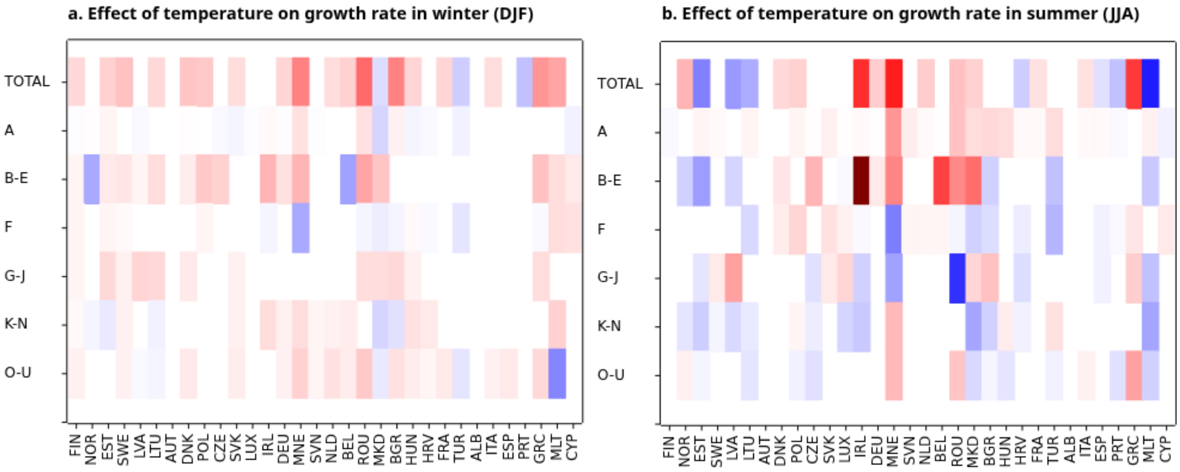
a. Effect of temperature on growth rate in winter (DJF)
b. Effect of temperature on growth rate in summer (JJA)
TOTAL
A
B-E
F
G-J
K-N
O-U
FIN NOR EST SWE LVA LTU AUT DNK POL CZE SVK LUX IRL DEU MNE SVN NLD BEL ROU MKD BGR HUN HRV FRA TUR ALB ITA ESP PRT GRC MLT CYP
FIN NOR EST SWE LVA LTU AUT DNK POL CZE SVK LUX IRL DEU MNE SVN NLD BEL ROU MKD BGR HUN HRV FRA TUR ALB ITA ESP PRT GRC MLT CYP

Reviewer 2

Summary

The paper examines the relationship between temperature and economic activity over the period 1980-2019, using NUTS3 (i.e. provincial) data for Europe. The temperature-growth relationship is analyzed through fixed-effect panel regression techniques, where the growth rate of annual gross value added (GVA) per capita is function of annual temperature (and precipitation). While the empirical literature typically models this relationship through a quadratic-in-temperature specification (as in Burke et al., 2015), the authors use spline regressions, which do not impose a predetermined polynomials to model non-linearities. Unlike most studies that find an inverted U-shape relationship for global economies, the results for Europe show a U-shape relationship between temperature and economic growth for initial temperatures between 0°C and 20°C degrees. Specifically, cold (hot) regions show a negative (positive) effect of temperature rise on GVA per capita growth. There is evidence of a beneficial (detrimental) impact of temperature shocks only at extreme temperatures, i.e. below 0°C degrees (above 20°C degrees). The authors also investigate potential transmission channels through which temperature affects economic growth, finding significant heterogeneity across provinces (both within and between countries) and economic sectors.

General comments

The topic of the paper is highly relevant and fits well within the growing literature on the temperature-growth nexus. The findings are particularly interesting, as they offer new insights into the complexity of the relationship between temperature and economic activity, moving beyond the traditional hot vs. cold region comparison. I like the idea of not relying on predetermined polynomials to model the non-linear relationship between temperature and economic activity (e.g., Burke et al., 2015, use a quadratic polynomial, Newell et al., 2021, rely on higher-order polynomials). The authors conduct rigorous robustness checks, showing that their results are not driven by data selection or model specification. Moreover, they provide compelling explanations for their findings, emphasizing the role of sectoral differences in shaping the temperature-growth relationship. However, there are a few areas I would recommend the authors to further explore.

RESPONSE:

Thank you for your positive response to the paper.

Comments

Contribution

B1) The Introduction is well-written and clear, but I would suggest emphasizing more explicitly the main contribution of the paper. Is the main innovation the empirical approach (i.e., the use of spline regressions), or does it lie in the use of more granular data at the NUTS3 level? As for the latter, a recent ECB working paper (i.e., Usman et al., 2024) also

examines the temperature-growth relationship using NUTS3 data for Europe. I suggest comparing your results with those of Usman et al. (2024).

RESPONSE:

We have emphasised the main contributions of the analysis which are:

1. the disentangling of the important heterogeneous effects that the more granular analysis affords (across space and economic sectors); 2. Our finding that the aggregate (global) relationship between temperature shocks and GDP, which exhibits an inverted U shape, is not robust in the European case.

Regarding the former, as we write in the paper (page 1):

> "[...] country level, economy wide temperature-economy relationships reflect the net effect of temperature on productivity across the constituent sectors of the economy and across different sub-national regions. In each case, important sectoral and geographic heterogeneity, for which there is evidence at the micro-level for outcomes such as wages, health,and recreation, is overlooked. The heterogeneity in the response of GDP to temperature shocks is however critical to inform efficient climate adaptation and mitigation policies".

We have also noted the ECB working paper by Usman et al 2024 and how our paper relates to their hypotheses and approach. In short, their approach uses the NUTS3 data to focus on the impact of extreme events at the **regional** level, using DID on local projections. We focus first on aggregate GDP effects for Europe, highlighting differences in the response function from preceding work, then on **district** level heterogeneity in the economic response to deviations in annual mean temperature from its long-term mean. This approach makes us comparable to previous work including the influential study by Burke et al. (2015) in terms of methods and research questions, and enables us to highlight important differences in the conclusions. The Usman paper is very interesting, but answers a separate question using this rich dataset.

Nevertheless, we have been clearer about the contributions of the paper in general and in light of this paper, adding the following paragraph to the end of the introduction:

> "The key contributions are the specification of a Europe-specific relationship between annual mean temperature (not extreme events per se, Usman et al. 2024) and aggregate income, then to illustrate the complex and granular heterogeneity of temperature effects by NUTS3 district and by sector. The findings indicate the unique structure of aggregate, spatial, and sectoral costs that can emerge from more granular analysis, and the need for bespoke climate adaptation strategies in Europe."

Empirical analysis

B2) It is not clear whether the f(.) functional is linear when the authors turn their focus on slope heterogeneity, by allowing temperature effect on growth to differ by district or by country (see pag. 8 in the econometrics frameworks section).

RESPONSE:

The function is linear-by-district. We have now made this clear in Figure 3 in which figures a and b are labeled as linear.

See also the revised fourth paragraph in the Methods section:

> "To examine heterogeneity of the estimated effects, we estimate models in which we allow for the effect of annual mean temperature to differ by district or by country. This means that the function f = [...] to obtain district-specific linear coefficients and f = [...] to obtain country-specific linear coefficients [...]. At the level of countries, we also estimate a quadratic-by-country model with f = [...]."

In essence, this specification implements a "locally-linear" approximation of the overall non-linear relationship between annual mean temperature and GDP growth rates in Europe. In other words, for each district/country, the relationship is assumed to be linear over the support of annual mean temperatures historically observed in that district/country. The variation in the estimated district/country specific relationships then allows for non-linearities over the full support of annual mean temperature observed in all of Europe.

For the by-country model, for which we have more observations for each spatial unit (in the above specification: district; here: country), we also estimate a non-linear (quadratic-by-country) version of the model. The aggregate costs are very similar to the linear-by-country model (SI Figure B6a).

B3) It would be helpful for the reader to use, in the econometric framework, footnote in table s3 to replace the description of how the authors compute projections.

RESPONSE:

We have followed this suggestion and have added the following sentence to the caption of SI Table B2:

> "The costs are quantified as changes in the average annual growth rate between the two periods and shown in percentage points."

This corresponds to the penultimate paragraph of the Methods section, which now has the following opening sentence:

> "For projections of the effect of climate change on GDP for the RCP4.5 scenario (2070-2099 versus 1985-2014), the costs are quantified as changes in the average annual growth rate between the two periods and shown in percentage points (see also SI Table B2). We use our estimated models to calculate the estimated annual growth effect [...] for every year of the historical and future period. We then subtract the average annual growth effect of the future period from the average annual growth effect of the historical period. We do this for each climate model separately and then calculate the median of the final cost estimate."

B3) (Part 2) Moreover, in the discussion of results, it would be helpful to describe how do these projections compare with findings in the literature for global economies (see for instance the recent paper by Bilal and Känzig (2024)

RESPONSE:

We have added a sentence to the discussion section, as suggested by the reviewer:

> "[...] Similarly, our focus on the response of local economies to local temperature shocks allows us to study heterogeneity in great detail, but we do not attempt to quantify any indirect costs of future climate change to a location due to its trading relationship with the rest of the world, which may be substantial (Bilal and Kaenzig 2024, Zappala 2023)".

B4) The authors estimate a static panel regression with no lagged temperature levels as additional regressors. However, the recent study by Nath et al. (2024) points at the importance of including lags of temperatures (and GDP) in the model specification, given the serial correlation typically observed in these series. Do the main results remain similar when lags of temperature and GDP are included?

RESPONSE:

We have undertaken this suggested analysis with lagged temperature levels as in Nath et al. 2024. The main results are robust to these models (see SI Figure B4 f,g,h, for convenience also inserted below).

We have added the following paragraph to the end of the Main Results section:

> "For robustness we estimate a model inspired by (Nath et al. 2024) with lagged temperatures, which yields very similar results (SI Figure B4)."

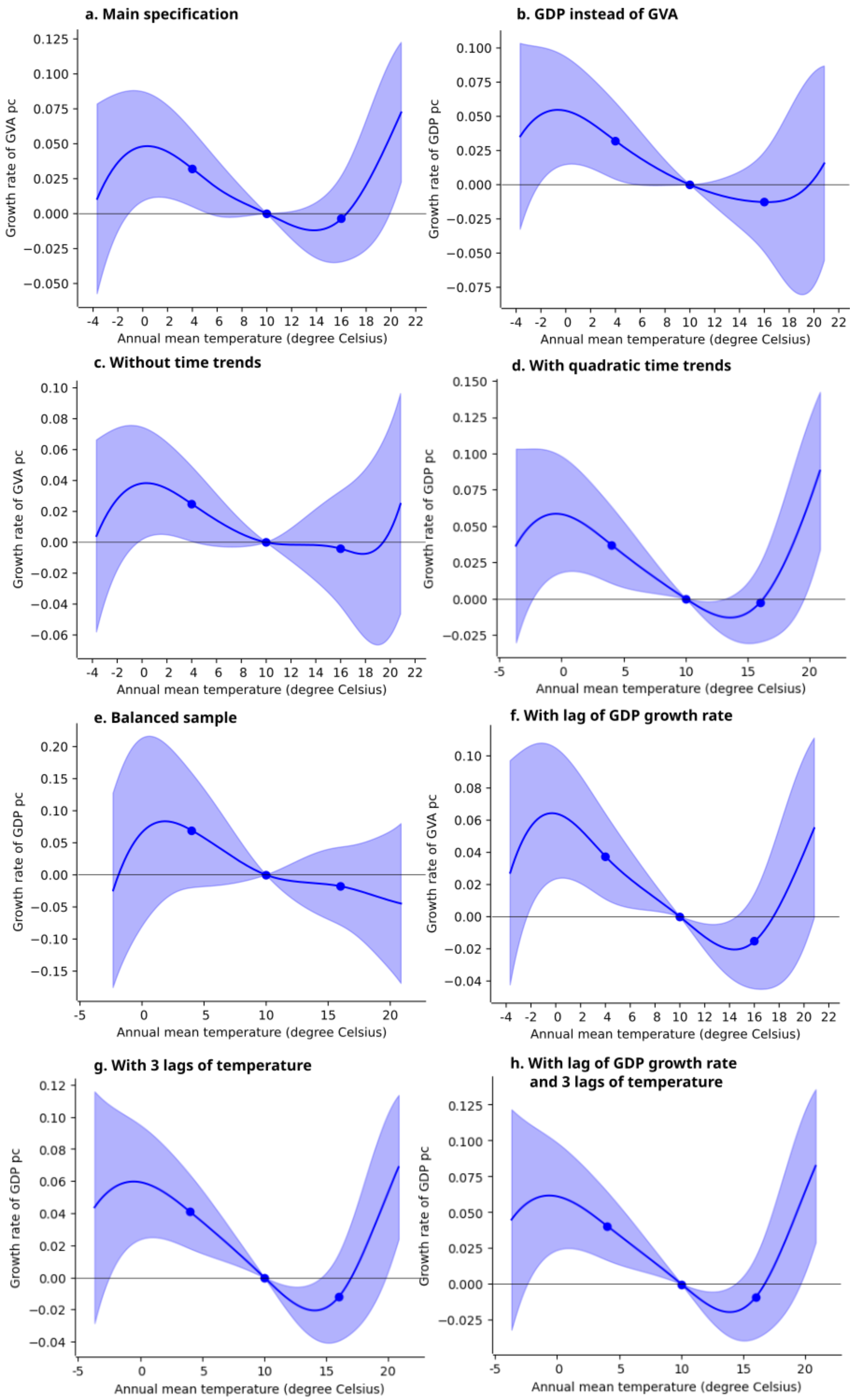
a. Main specification
Growth rate of GVA pc
Annual mean temperature (degree Celsius)
b. GDP instead of GVA
Growth rate of GDP pc
Annual mean temperature (degree Celsius)
c. Without time trends
Growth rate of GVA pc
Annual mean temperature (degree Celsius)
d. With quadratic time trends
Growth rate of GDP pc
Annual mean temperature (degree Celsius)
e. Balanced sample
Growth rate of GDP pc
Annual mean temperature (degree Celsius)
f. With lag of GDP growth rate
Growth rate of GVA pc
Annual mean temperature (degree Celsius)
g. With 3 lags of temperature
Growth rate of GDP pc
Annual mean temperature (degree Celsius)
h. With lag of GDP growth rate
and 3 lags of temperature
Growth rate of GDP pc
Annual mean temperature (degree Celsius)

B5) Several studies have shown that temperature shocks affect economic activity through changes in labour productivity (see, e.g., Acevedo et al., 2020; Mohaddes et al., 2024, among others). What role does labour productivity play in your analysis? Furthermore, it would be interesting to explore also how investments in different sectors respond to rising temperatures.

RESPONSE:

This is an extremely interesting point. We have added references to these papers in the discussion of the paper having taken the decision that undertaking a micro study in our case would require a different data set entirely on the economic side. This is fertile ground for future work. In terms of thinking through the mechanisms this is an incredibly helpful comment and we have written the following text in the paragraph just before the conclusion where we discuss mechanisms.

> "For these and related reasons, a number of studies have shown that temperature shocks also affect activity through labour productivity (see, e.g., Acevedo et al., 2020; Mohaddes et al., 2024). Further microeconometric research to explain how these potential mechanisms translate into the precise pattern of spatial and sectoral heterogeneity would be a fruitful next step in the analysis."

B6) Why do the authors use a quadratic polynomial for precipitations, while they use cubic splines for temperature? Is this choice motivated by evidence in the empirical literature?

RESPONSE:

The choice of specification for these two physical weather/climatic determinants of GDP simply follows the literature to date and the extent of robustness that is typically performed. We have undertaken further robustness on the recommendation here. Below we see the cubic spline analysis of precipitation, to which the central aggregate results are robust. We note this in the methods section at the end of para 3 of the "Econometric framework" section.:

> "We also undertake the analysis with a cubic-spline analysis for precipitation. The results remain robust and we do not report them here."

We hope that answers the point raised here by the referee. We would like to point out that we also undertake extensive robustness tests with in total 36 different measures of precipitation as control variable, including measures of extreme precipitation, effectively also testing robustness of our main results to possible non-linear effects of rainfall that could possibly be correlated with temperature shocks (SI Figure B5).

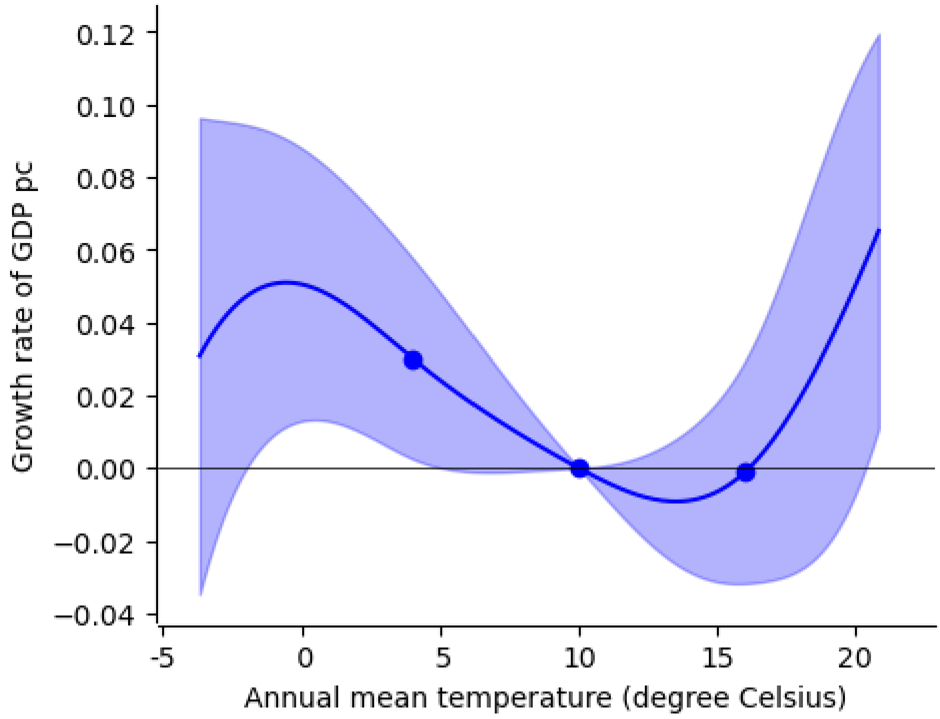

B7) The authors should define, in the Conclusions, the limitation of their study (for instance, labor productivity and investments are transmission channels which have not been explored) suggesting future scope of research

RESPONSE:

In addition to the point made prior to the conclusion in response to point B5 we have rewritten the last paragraph of the conclusion as follows:

> “The paper illustrates that care is needed in evaluating the impacts of temperature change and drawing broad conclusions about how the costs and benefits of climate change are distributed across different geographies. Considering Europe separately from the rest of the world has provided important new insights. Future research can further illuminate the costs of climate change in cold regions of the world outside Europe. This will add more evidence on the mechanisms through which warmer-than-average years can reduce economic production in certain industries. Furthermore, using dis-aggregated data allowed us to study geographic and industry heterogeneity in great detail, but the relatively few years of observations prevent us from studying the dynamics and persistence of temperature shocks, which future research may further explore building on the existing literature (31, 32, 35, 36). Similarly, our focus on the response of local economies to local temperature shocks allows us to study heterogeneity in great detail, but we do not attempt to quantify any indirect costs of future climate change to a location due to its trading relationship with the rest of the world, which may be substantial (37, 38). Other research questions arising from our results concern the possibilities of adaptation to gradual changes of temperature in the long-run, which might be underestimated or overestimated in our results. The role of specialisation, and the spatial and sectoral linkages through

which temperature effects operate, are also obvious avenues for future research. While we have demonstrated the heterogeneous effects across space and sectors, we are limited in what we can say about the precise mechanisms that lead to the spatial and sectoral pattern we observe. As more granular data becomes available, new insights into the challenges of climate change can be gained from the geographically explicit analysis found in this paper, and a broader evidence base developed to inform policy. If the objective is to better understand the impact of climate change, and hence better prepare and adapt, mechanisms relating to labor productivity, investment, the response of infrastructure and health appear to be primary areas of interest for future research. Future research should also bear in mind that GDP and GVA do not capture all of the impacts of climate change on long-term human well-being or the biosphere."

Minor comments

B8) I suggest adding notes below the figures and tables reported in the Appendix.

RESPONSE:

We have expanded the captions of figures and tables reported in the SI, including also information typically found in notes below figures and tables. In many cases, we refer to the Methods section of the manuscript for more detailed information, which we have expanded too. We will be happy to add additional information to the figures and tables in the SI if requested by the reviewer.

References

- Acevedo, S., Mrkaic, M., Novta, N., Pugacheva, E., & Topalova, P. (2020). The Effects of Weather Shocks on Economic Activity: What are the Channels of Impact? Journal of Macroeconomics, 65, 103207.
- Burke, M., Hsiang, S. M., & Miguel, E. (2015). Global non-linear effect of temperature on economic production. Nature, 527, 235-239.
- Bilal, A., & Känzig, D. R. (2024). The Macroeconomic Impact of Climate Change: Global vs. Local Temperature. NBER Working Paper No. 32450, National Bureau of Economic Research.
- Mohaddes, K., Ng, R. N. C., Pesaran, M. H., Raissi, M., & Yang, J.-C. (2023). Climate change and economic activity: evidence from US states. Oxford Open Economics, 2, odac010.
- Nath, I. B., Ramey, V. A., & Klenow, P. J. (2024). How Much Will Global Warming Cool Global Growth? NBER Working Paper No. 32761, National Bureau of Economic Research.
- Newell, R. G., Prest, B. C., & Sexton, S. E. (2021). The GDP-Temperature relationship: Implications for climate change damages. Journal of Environmental Economics and Management, 108, 102445.
- Usman, S., Fernandez, G. G. T., & Parker, M. (2024). Going NUTS: the regional impact of extreme climate events over the medium term. ECB Working Paper Series No. 3002, European Central Bank.

Response to Reviewer #1:

A1) The authors have addressed the reviewers' primary comments.

The results are still somewhat counter-intuitive based on some of the existing literature. In particular, it would be helpful for the authors to compare their findings to "Seasonal Temperatures and Economic Growth in the United Kingdom" by Lotanna E. Emediegwu, Veronica Vienne, and Jubril Animashaun in the Oxford Bulletin of Economics and Statistics, which find different seasonal patterns of effects in the UK.

Can you explain why your results differ?

Response: We appreciate the reviewer's suggestion to compare our findings with this earlier work. There are two major methodological differences between our paper and this earlier work. First, we use economic data at the level of NUTS regions , (including for the UK), whereas the authors use data at the level of local authorities, which is more granular.We adopt the NUTS classification to ensure consistency and comparability across all European regions. Second, we estimate a "linear-by-season" model, whereas the authors estimate a "quadratic-by-season" specification, in which they include second-order polynomials of seasonal temperature in the regression.

When we estimate a quadratic-by-country model that is identical to their model but with our data we get qualitatively similar results (see the table below). To further ensure comparability and clarify any remaining differences, we reached out to the authors to inquire about a replication package. Unfortunately, such materials were not available. We hope this additional robustness removes any concerns.

	Estimated coefficient, standard error, and significance	
Variable	Emediegwu et al.<br>(Table 2, Column 1)	Our estimates
T_DJF	1.128*** (0.180)	0.219** (0.109)
T_MAM	−0.837** (0.383)	-2.513*** (0.079)
T_JJA	−1.148** (0.551)	-1.694*** (0.252)
T_SON	−1.172*** (0.345)	-1.209*** (0.112)

Also, please note that in the paper we already show the robustness of our main results for using either "linear-by-country" specifications and "quadratic-by-country" specifications (see SI Figure B6 and SI Table B2). In addition, we have added a sentence in the paper motivating additional, country-specific analysis that uses for each country the best available economic data and examines industry-specific and season-specific effects:

“Detailed analysis of individual countries, especially disaggregated by industry and season (e.g. Colacito et al. 2019, Emediegwu et al. 2025), can complement regional and global analysis.”

Response to Reviewer #2:

The authors carried an extensive revision of the manuscript addressing some of the issues raised. The following are my comments:

B1) As for the issue of temperature seasonality, it would be useful to clarify which months are included in winter and which one in summer? It would be interesting to know whether the authors aggregate six months for each season, since, in this case, they won't be able to capture further observations concentrated in the distribution tail which, I guess, might corroborate the evidence of non-linearities in temperature-growth relationship discovered for Europe.

Response: We use the meteorological definitions of the two seasons: winter = DJF, summer = JJA. This was stated in the text of the manuscript, but not in the response to the reviewer’s comments. We apologise for this oversight.

B2) As for the specification with lags in the dependent variable and in temperature, the authors should fully account the Nath et al (2024) insights:
a) if there is any evidence of serial correlation in temperature, then it would be worthwhile to filter out the anticipated component of temperature and identify the shock to temperature and to explore its effect on growth.
b) more importantly, the focus on a panel (with long time series) allows to draw conclusions on growth vs level effect (Nath et al, emphasize persistence effects as an intermediate case). In other words, the authors, in the revised draft, control for lags in temperature and growth (dependent variable) but they only report the impact coefficients (there might be evidence, in line with other panel studies, of sign reversals over medium term horizons). Therefore, it would be useful to know whether the non-linear model specification holds for horizons beyond one year.

Response:

a) We appreciate the author’s interest in the dynamic effect of temperature on economic growth. We are aware that there is an ongoing debate on the dynamic attributes of the effect of temperature on economic growth in the literature.

The distinction between an “anticipated” component and a “surprise” component of temperature, as introduced in the working paper by Nath et al. (2024), is a novel contribution of that paper, and we understand that it is grounded in econometric theory.

We would like to point out that our contribution is a different one - we focus on Europe and study regional and sectoral heterogeneity. Our main reference is the earlier published and very influential work on the effect of temperature on economic growth, especially Burke et al. (2015). In order to be able to compare our results to theirs, and to focus on our main

contribution concerning heterogeneity, we do not think it is helpful to add additional methodological novelties that have not yet been published in a peer-reviewed journal.

Furthermore, in our setting with the local GDP data in Europe, we are using a wide (the number of districts is 1494) but a short panel (the number of years with data is between 0 and 40, SI Figure A4). Our setting is therefore well suited to study cross–sectional heterogeneity, but less suited to study the dynamics proposed.

However, we understand that Nath et al. (2024) make an important point about possible biases due to time series properties of both temperature and GDP. For this reason, we have implemented the methodology of Nath et al. (2024) despite the above mentioned caveats. We proceed as Nath et al. (2024): in the first step, we regress annual mean temperature on its own lags, letting the coefficients of the lags vary by country and including year and district fixed effects as in Equation 4 in Nath et al. (2024). In the second step, we regress GVA growth rates on the residuals of the first regression, which can be interpreted as the “surprise” component of temperature.

When doing so, we follow Nath et al. (2024) also by interacting this “surprise” shock with a time-invariant moderator variable. Nath et al. (2024) use a linear term of the climatic average temperature of a country as moderator. To allow for more flexibility, we use dummies for different intervals of the climatic average temperature of districts in our sample. This allows us to test whether the non-linear shape of the response of GVA to a temperature shock of our main specification is robust to using only surprise shocks. The results are shown in the following. The estimated coefficients can be interpreted as marginal effects over the given support. Their signs and magnitude can thus be compared to the main specification shown in SI Figure B1b. Overall, we find essentially the same pattern of marginal effects for the original temperature time series as for its surprise component.

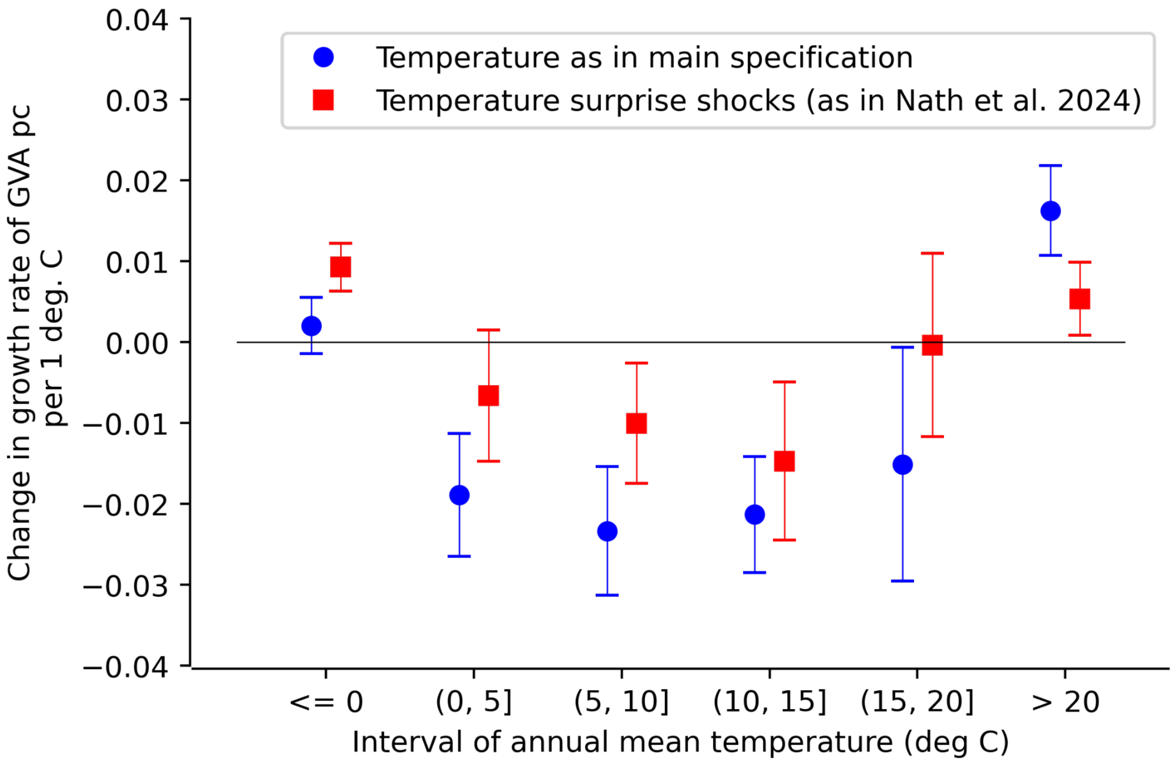

We have added a sentence on this in the manuscript:

> *"In another robustness check, we use only the "surprise component" of the temperature time series (Nath et al. 2024) and again recover essentially the same non-linear effects across Europe (SI Figure B6)."*
> *."*

We also briefly explain the methodology in the Methods section.

b) We agree with the reviewer that the question of the "persistence" of the effect of temperature on economic growth is the more important question. The same limitations that we pointed out in our response to a) apply, but we agree that it is an important question whether the heterogeneity that we find also holds true for the time-cumulative effect.

In the following we show the cumulative marginal effect of a warmer-than-average year on economic growth for models with 0, 2, and 5 years of lags of temperature. The choice of lags is motivated by Burke et al. (2015) SI Table S2. We show the results for our baseline specification (without the lagged dependent variable) and for the dynamic specification that the reviewer suggested (with a lagged dependent variable). For parsimony of the model, we use a quadratic polynomial specification as in Burke et al. (2015) and in SI Figure B2 c.

The results below are also shown in the new SI Figure B6. The numerical results can be found in SI Table B2.

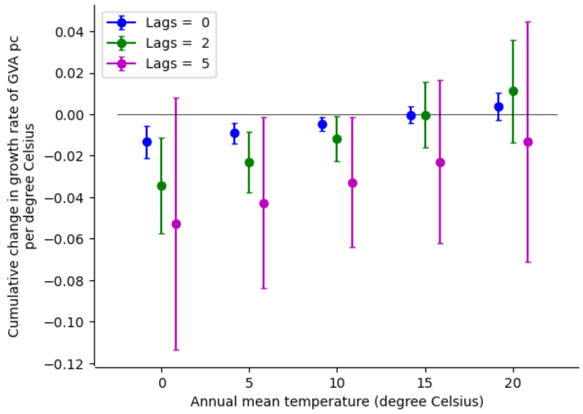

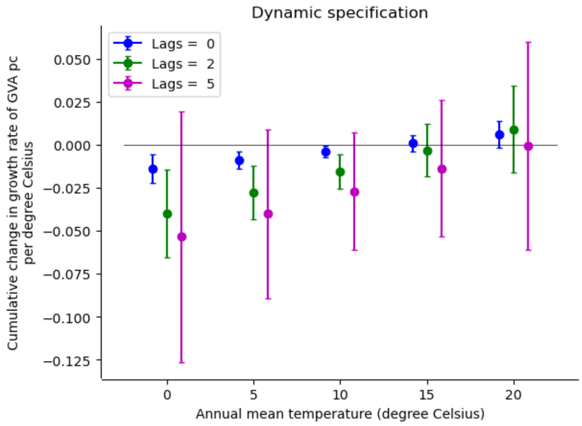

The results show that for relatively cold temperature levels (0, 5, and 10 degrees Celsius), the instantaneous effect is negative and the cumulative effect is even more negative. For 0 degrees Celsius, the cumulative effect is also negative and large but statistically insignificant.

For relatively warm temperature levels (15 and 20 degrees Celsius), the instantaneous effect is insignificant and positive, while the cumulative effect is insignificant and negative.

This shows that the main result that warming is detrimental for GDP in cold places also holds true for the cumulative effect. Furthermore, the results also suggest that the possibly positive effect of warming in warm places may be only temporary. These results are all in line with the existing narrative that focuses only on the instantaneous effect.

B3) The slope heterogeneity (across districts) is controlled through panel linear regression with the slope coefficients varying across districts. It is not clear whether, in this case, the authors estimate by OLS a time series regression for each district or if they pool the data and include fixed (cross sectional, time fixed effect, and heterogeneity in the slope coefficients). The second model has a proliferation of parameters which might require to resort to some shrinkage estimation methods. The first specification can be recast within the Mean Group approach and it can be handled by OLS and then the authors can average across different groups (districts with temperature below 0 Celsius degree, etc...)

Response: In our main specification, and for all results presented in the previous version of the manuscript, we pool the data and include an interaction of the unit fixed effects with the temperature variable. This allows us to estimate heterogeneous “slopes”, as the reviewer writes. This approach allows us to compute valid standard errors clustered at the level of countries.

The reviewer is correct that the model therefore has a lot of parameters that need to be estimated, which may raise concerns about the stability of the estimated parameters. In response to the comment and to address this concern, we have additionally implemented the alternative approach pointed out by the reviewer: we estimate the model separately for each unit. This has the disadvantage that we cannot account for spatial autocorrelation of

the residuals when calculating the standard errors. Nevertheless, we think it is a worthwhile exercise.

We check the robustness of our main results on the heterogeneity of estimates across districts by comparing the results of the two approaches. To do so, we calculate the correlation across districts between the estimates obtained with the first approach and the estimates obtained with the second approach. The correlation coefficient is 0.91. Concerns about parameter stability in our main specification appear therefore only minor in our setting.

References

- Nath, I. B., Ramey, V. A., & Klenow, P. J. (2024). How Much Will Global Warming Cool Global Growth? NBER Working Paper No. 32761, National Bureau of Economic Research.

Response to Reviewer #1:

Reviewer #1 (Remarks to the Author):

The authors have addressed the reviewers' concerns by estimating additional models. There is the potential for future research to expand on this paper but that is beyond the scope of this paper.

Response: We thank the reviewer for their constructive comments.

Response to Reviewer #2:

Reviewer #2 (Remarks to the Author):

The authors have addressed most of the issues I raised.

Response: We are glad to read that most issues have been addressed.

A1) As for my comments B2 b) I have the following remarks.

The authors stress that their contribution is with respect to Burke et al (2015) and they add that they do not think it is helpful to add additional methodological novelties that have not yet been published in a peer-reviewed journal. However, I argue, the literature on a (non-linear) relationship between temperature and output has since then produced several peer-reviewed journal publications which focus not only on contemporaneous linkages, but on dynamic linkages and on the level vs growth effect of temperature. As I commented in my previous referee report, the NBER working paper (which is a prestigious publication, subject to peer review) by Nath et al, contributes to the debate growth vs level effect, taking into account persistence in both temperature and output. My suggestion, then, is to include full dynamics and, in particular, to explore not only a contemporaneous but also a relationship beyond one year horizon. Although the authors follow my suggestion (see B2 a of my previous report) and they estimate temperature shocks, I guess that the model specification producing the results in Fig 6 in the supplementary material and also in the rebuttal letter is static (e.g. the authors explore only the contemporaneous impact of a temperature shock on growth). Therefore: 1) Can the authors provide an equation, at least, in the supplementary material describing the model specification underlying Fig 6?

Response: We have added the Equation of SI Figure B6 to the Methods section of the manuscript. We provide a brief summary here.

Our main model can be written as (see Methods section for a definition of each term):

$$g_{i,y} = f\left(T_{i,y,d};\beta\right) + h\left(P_{i,y,d};\gamma\right) + \alpha_i + \theta_y + y\xi_i + \epsilon_{i,y} \qquad (1)$$

In SI Figure B6, we show the results of a model where the first term is defined as:

$$f = \sum_k \delta_k T_{i,y} \beta_k \quad (2)$$

The model accounts for the moderating effect of the mean climate on the effect of temperature shocks by interacting the temperature variable $T_{i,y}$ with dummies for intervals of climatological annual mean temperature, δ_k. This is motivated by the non-linearities (heterogeneous effects of temperature) that we report throughout the paper. It is a very flexible model that does not make strong parametric assumptions.

We estimate the model once with annual mean temperature $(T_{i,y} = \overline{T}_{i,y}$ in Equation 2) and once with temperature "surprise shocks" ($T_{i,y} = \tilde{T}_{i,y}$ in Equation 2). The surprise component is defined as in Nath et al. (2024) as the residual of an autoregressive model:

$$\overline{T}_{i,y} = \sum_{l=1}^{3} \beta_{l,c(i)} \overline{T}_{i,y-l} + \alpha_i + \theta_y + \tilde{T}_{i,y} \quad (3)$$

where we allow the coefficients of the lags to vary by country to allow for regional heterogeneity in the dynamics of temperature.

A2) In general, to my understanding, all the figures produced in the main text and in the supplementary material are on the contemporaneous (one year) relationship between output and temperature (or temperature shocks). This should be explicitly stated in the main text.

Response: The reviewer is correct that the paper primarily focuses on the contemporaneous effect of a temperature shock on the growth rate in the same year.

In additional analysis, we also show cumulative effects. Earlier versions of the SI already included cumulative effects in SI Table B1 ("Cumulative marginal effects"). In the previous revision we added SI Figure B7 and now in this revision we added SI Figure B8, which also shows cumulative effects of temperature on economic growth.

For additional clarity, we have added the definitions of the "contemporaneous effect" and the "cumulative effect" to the Methods Section of the paper:

$\frac{\partial g_{i,y}}{\partial T_{i,y}}$ and $\sum_{y=0}^{Y} \frac{\partial g_{i,y}}{\partial T_{i,0}}$, respectively.

We agree that the focus on the contemporaneous effect can be further emphasised in the main text. We have added this information prominently in the abstract:

> "[...] Focusing on Europe, we use administrative district level data on the growth rate of Gross-Value Added (GVA) and GDP to estimate the contemporaneous impact of temperature on economic growth at the national, district, and industry levels. [...]"

We have also added it to the last paragraph on the introduction in which we describe our methodology:

> "[...] We generally focus on the contemporaneous effect of temperature shocks on growth rates in the same year. [...]"

We also describe the results for the cumulative effect in the manuscript (at the end of the first paragraph on page 3):

> "[...] Additional analysis suggests that the drop in GVA is persistent in cold regions, but the cumulative effect is similar to the contemporaneous effect somewhat sensitive to the model specification in warm regions (SI Table B1; SI Figure B7)."

The focus on the contemporaneous effect is then more extensively discussed at the end of the manuscript (see also our response below to comment A6).

A3) The only exceptions are in the rebuttal letter which shows a Figure on "Cumulative Change in growth rate of GVA pc per degree Celsius". These figures have with two panels: the right hand side named "Dynamic Specification" and the left hand side which, I guess, it is based on a non-dynamic specification which, however, is the only one showing up in the supplementary materials (see Fig B7 in SI). Therefore: 2) Can the authors provide an explanatory equation (and the associated code to reproduce the results) describing the Figures in the aforementioned two panels and, in particular, the one for the dynamic specification (which is the one I asked for in my previous report)? I could not find such explanatory equation in the main text nor in the supplementary material.

Response: We apologise for the missing equations. We have added the equations to the Methods section of the paper. We provide a brief summary here.

The reviewer is referring to SI Figure B7a, which was SI Figure B7 in the last version, and which was the subfigure on the left hand side of the figure that we included in our response document. The figure shows the cumulative marginal effect for a second-order polynomial model, as the reviewer points out. For this figure, we estimate the model in Equation (1) with:

$$f = \overline{T}_{i,y}\beta_{1)} + \left(\overline{T}_{i,y}\right)^2 \beta_2 \quad (4)$$

For the cumulative effects, we estimate the model of Equation (1) with (4) with additional *L* lags of temperature (a "distributed lags model"):

$$f = \sum_{l=0}^{L} \left(\overline{T}_{i,y-l}\beta_{1,l} + \left(\overline{T}_{i,y-l}\right)^2 \beta_{2,l}\right) \quad (5)$$

and use the coefficients of Equation (5) to calculate the cumulative marginal effect at different temperature levels $\overline{T}$ as

$$\sum_{l=0}^{L} \left(\beta_{1,l} + 2\overline{T}\beta_{2,l}\right) \quad (6)$$

The dynamic models include a lagged dependent variable. We would like to note that estimates of this specification are affected by the Nickell bias, which is why we are generally cautious in interpreting the results of dynamic specifications, especially given the short time dimension of our panel. The version with lagged dependent variable reads:

$$g_{i,y} = f\left(T_{i,y,d};\beta\right) + h\left(P_{i,y,d};\gamma\right) + \alpha_i + \theta_y + y\xi_i + \rho g_{i,y-1} + \epsilon_{i,y} \quad (7)$$

The contemporaneous effect of the dynamic models is calculated in the same way as the contemporaneous effect of the static model, but the cumulative effect of the dynamic model accounts for the propagation of growth shocks through the lagged dependent variable. We refer to this cumulative effect in the Methods section now as *dynamic cumulative effect* and calculate it by multiplying the cumulative effect by the limit of the geometric series:

$$\frac{\sum_{l=0}^{L}\left(\beta_{1,l} + 2\overline{T}\beta_{2,l}\right)}{1-\rho} \quad (8)$$

The results are shown in SI Figure B7 and further below in response to comment A5.

A4) Moreover, the codes needs a README file to see the different model specifications including those producing Figures B6 and B7 (especially how you compute the compute the cumulative marginal effects in Fig B7, SI).

Response: We apologise that the instructions in the README file in the previous version of the replication package were not clear. We have expanded the README file, including also a section that indicates in which script a figure is generated. Furthermore, we have added additional comments to the top of each script.

The code is available here: https://github.com/nykcrj/europe. The data are available here: https://tinyurl.com/268x89r8 .

See our response to A3 for the equations of the cumulative marginal effects. The calculation is implemented in the script *03_cumulative.py* .

A5) As long as the results in Figure B7, SI, are associated with the outcome of a dynamic panel regression (please specify an equation describing the model and how you compute the cumulative marginal effects), the empirical findings suggest that only in cold countries there is evidence of a statistically significant negative effect of temperature for an horizon up to two years ahead (a five year horizon implies statistically insignificant empirical findings regardless of temperature levels). In particular, in Fig B7 (SI) the authors use a dynamic model specification which is a quadratic polynomial. Therefore: 3) Is there any particular reasons why the authors do not extend the dynamic model specification also to cubic splines (they focus only on the quadratic model of Burke et al (2015)) since it is the main model specification producing Fig B1,B2,B4 and B5 in the supplementary material beyond Fig 1a in the main text?

Response: We have above included the equation of the dynamic model (Equation 7) and the equation of the cumulative marginal effect for the static model (Equation 6) and for the dynamic cumulative effect of the dynamic model (Equation 8).

In the previous version of the manuscript we showed the cumulative effect only for a second-order polynomial model because adding temporal lags to the model multiplies the number of explanatory variables in the regression. We chose the polynomial model as the most parsimonious model that still produces our main results (see SI Figure B2). Furthermore, the

choice of the second-order polynomial model means that we essentially replicate the analysis of persistence in Burke et al. (2015), see their SI C.2 “Growth versus level effects”, especially SI Table S2, so our results show as clearly as possible whether any differences from this earlier study can be explained with different persistence of the effects. This does not seem to be the case; we find similar non-linear patterns for the cumulative effect as for the contemporaneous effect, especially for cold temperatures, with less significance.

We agree with the reviewer that one may also want to use a more flexible model to investigate whether focusing on the cumulative effect substantively changes the non-linear relationship between temperature and growth that we report for Europe. For this reason, we have now added another analysis where we use a more flexible “linear-by-interval” model (see also Equation 2 above):

$$f = \sum_{l=0}^{L} \sum_{k} \delta_k \overline{T}_{i,y-l} \beta_{k,l} \quad (8)$$

This model is parsimonious and the cumulative marginal effect can be calculated as for a linear polynomial model. For each interval k we calculate (see also Dell et al. 2012):

$$\sum_{l=0}^{L} \beta_{k,l} \quad (9)$$

and for the dynamic model:

$$\frac{\sum_{l=0}^{L} \beta_{k,l}}{1-\rho} \quad (10)$$

The contemporaneous effects and the cumulative marginal effects are shown in the now extended SI Figure B7, for convenience also inserted below. We find that the *cumulative* marginal effects have the same sign (negative) as the *contemporaneous* marginal effects for relatively low climatological temperature (< 15 degrees C for the quadratic model, < 20 degrees C for the linear-by-interval model) with generally larger magnitude and larger standard errors (lower statistical significance) as we add lags to the model. For relatively high climatological temperatures, the results are ambiguous. For the quadratic model, we do not find significant effects. For the linear-by-interval model we find positive cumulative marginal effects with again decreasing statistical significance as we add lags to the model.

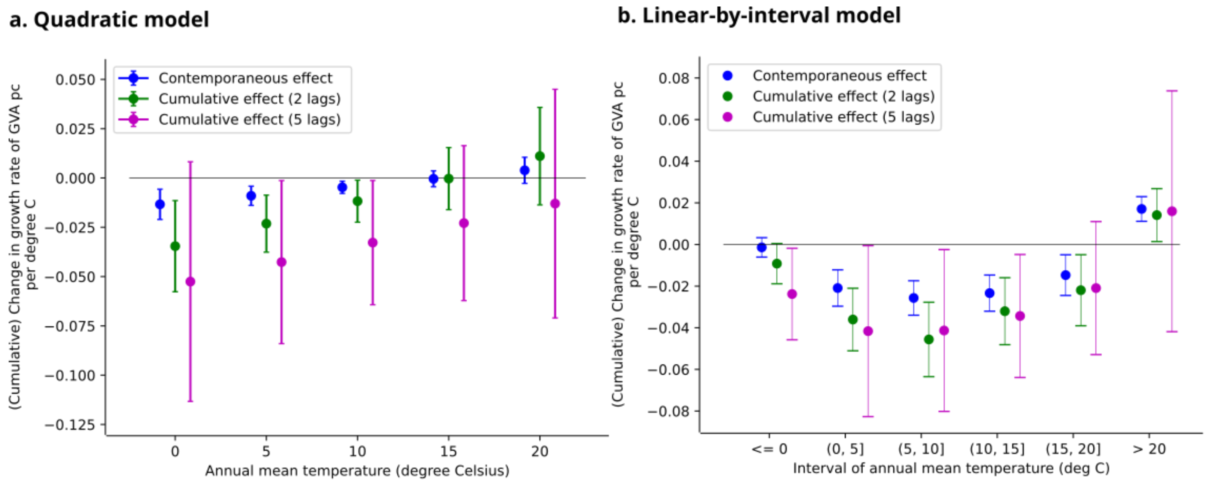

For the dynamic model, the contemporaneous effects and the dynamic cumulative effects are shown in SI Figure B8 and for convenience inserted below. The results are optically almost indiscernible from the results of the static model. This can mostly be explained with the low persistence of economic growth rates in our data (the estimated coefficient of the lagged growth rate is about -0.06).

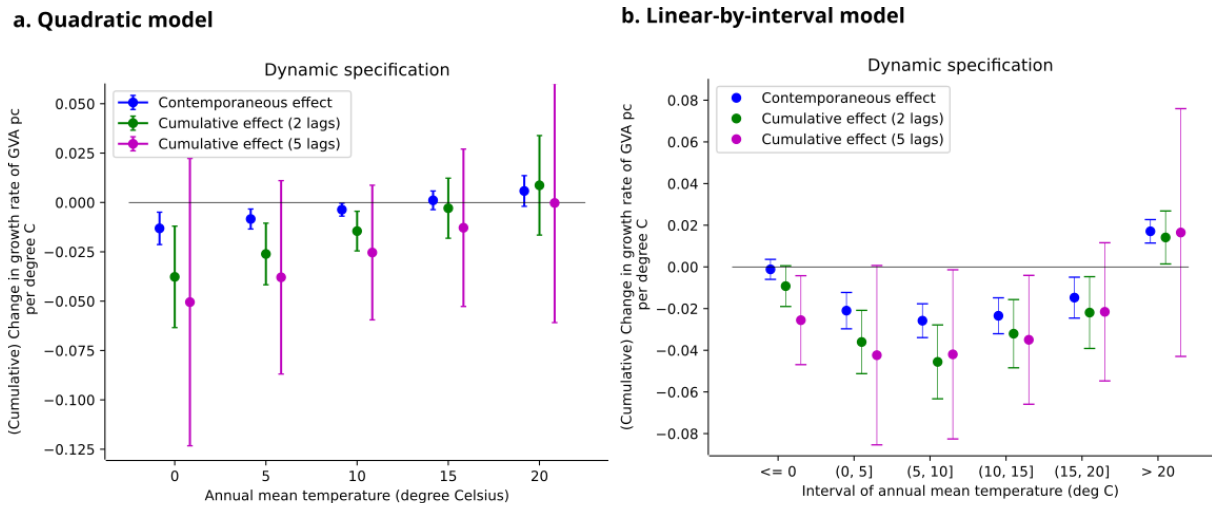

A6) Can the authors extend the dynamic model specification also to further robustness checks such as those for marginal effects by industry and by country (Fig 4 in the main text)?

Response: We have produced Figure 4 now also for a dynamic model specification. The results are shown in SI Figure B11. We have added Figure 4 and SI Figure B11 for easier comparison also below.

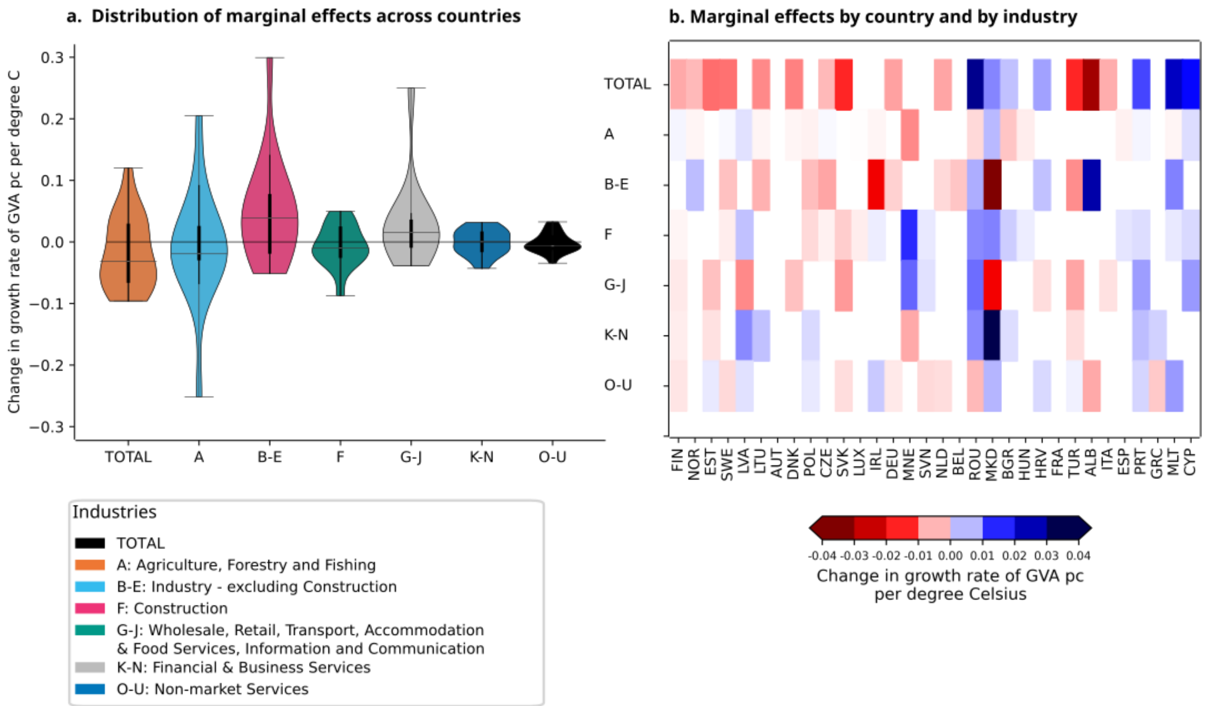

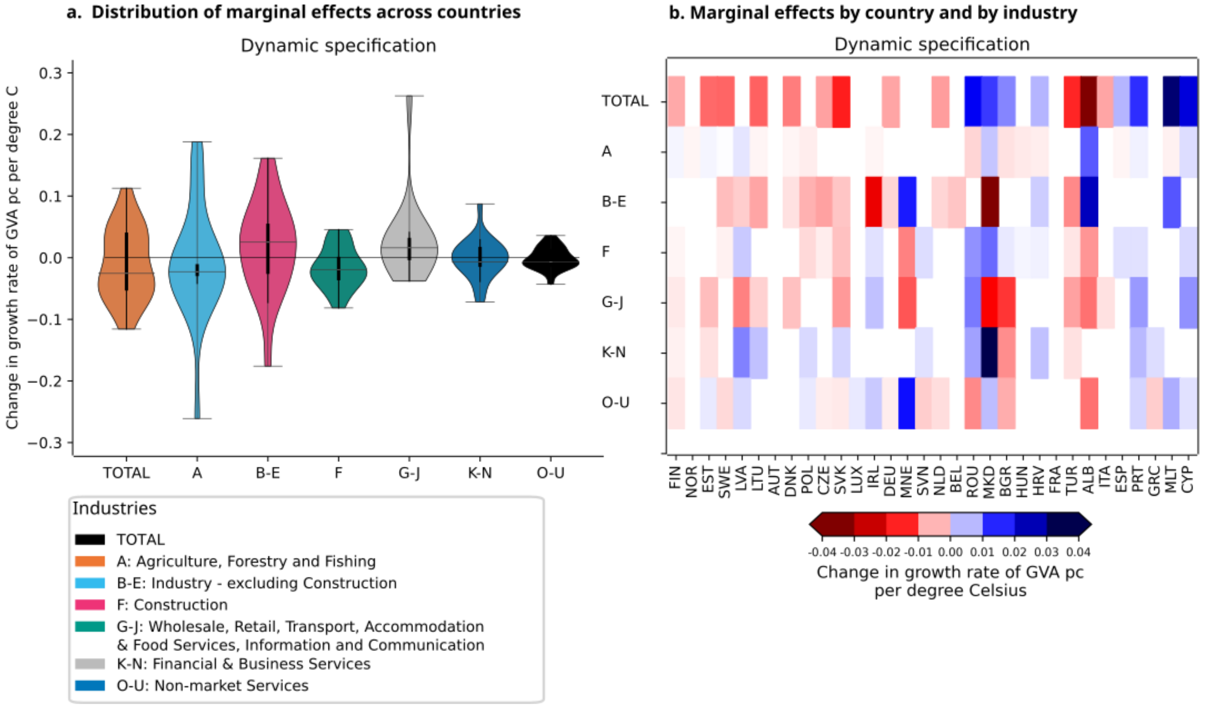

The results are again very similar with only very minor differences that do not change our interpretation of the results.

We agree with the reviewer that it makes sense to include some robustness test in the paper that provide assurance that our results and especially their discrepancy from earlier work, including Burke et al. (2015), is not due to e.g. different persistence of temperature shocks in Europe versus elsewhere. We have added all such results that the reviewer already requested in previous rounds and we have added additional results in this revision. However, we would also like to reiterate that our paper focuses on the spatial heterogeneity, not the temporal dynamics. The scope of our paper is already quite extensive and has

further grown during the review process. This includes several additional robustness tests proposed by the reviewer in earlier rounds. Furthermore, our data is rich in the spatial dimension (with more than 1000 districts) but short in the time dimension (with on average only 15 years of data). This means that any temporal dynamics can only be relatively poorly studied and will primarily add noise to our results. Other papers with other data (at the national level) that are less rich in the spatial dimension but that have longer time series - such as Nath et al. - should and do study the dynamics.

In response to the reviewer's comments, we have refined the discussion of this in our paper and made it a separate paragraph to give it more emphasis:

> "The paper illustrates that care is needed in evaluating the impacts of temperature change and drawing broad conclusions about how the costs and benefits of climate change are distributed across different geographies. Considering Europe separately from the rest of the world has provided important new insights. Future research can further illuminate the costs of climate change in cold regions of the world outside Europe. This will add more evidence on the mechanisms through which warmer-than-average years can reduce economic production in certain industries. Furthermore, using dis-aggregated data allowed us to study geographic and industry heterogeneity in great detail, but the relatively few years of observations prevent us from studying the dynamics and persistence of temperature shocks. Our robustness tests suggest that the non-linear patterns are similar for cumulative effects. Future research may further explore the temporal dynamics and persistence building on the existing literature (Acevedo et al. 2020, Bastien and Moore, 2022, Mohaddes et al. 2023, Nath et al. 2024)."

Reviewer #2 (Remarks on code availability):

As specified in the referee report, it would be more more helpful to have a README file

Response: The replication package includes a README file. We have expanded the README file as well as the comments in each individual script. See also our responses to A4 (including the link to the replication package).

References

- Nath, I. B., Ramey, V. A., & Klenow, P. J. (2024). How Much Will Global Warming Cool Global Growth? NBER Working Paper No. 32761, National Bureau of Economic Research.

- Dell, Melissa, Benjamin F. Jones, and Benjamin A. Olken. "Temperature shocks and economic growth: Evidence from the last half century." American Economic Journal: Macroeconomics 4.3 (2012): 66-95.
